# The effect of dexmedetomidine on emergence delirium of postanesthesia events in pediatric department: A systematic review and meta-analysis of randomized controlled trials

**DOI:** 10.1097/MD.0000000000039337

**Published:** 2024-09-06

**Authors:** Sunyu Tang, Jikai Liu, Zheng Ding, Ting Shan

**Affiliations:** a Wuxi Medical College, Jiangnan University, Wuxi, China; b Department of Anesthesiology, Jiangnan University Medical Center, Wuxi, China; c Department of General Surgery Department, Jiangnan University Medical Center, Wuxi, China.

**Keywords:** dexmedetomidine, emergence delirium, meta-analysis, pediatric

## Abstract

**Background:**

Emergence delirium (ED) is a common occurrence in pediatric postanesthesia events, leading to negative outcomes. Dexmedetomidine (DEX), as an anesthesia adjuvant, has shown promise in preventing ED in adult surgeries, and it has been increasingly used in pediatric surgical settings. However, its effectiveness in other postanesthesia events, such as MRI examinations and ambulatory surgery centers, remains unclear. This meta-analysis aims to assess the safety and efficacy of DEX in preventing ED in various pediatric postanesthesia events beyond surgery.

**Methods:**

Prospective randomized controlled trials were searched in Pubmed, Web of Science, and EBSCO until October 13, 2023. Comparisons were made between DEX and other sedatives or analgesics in different postanesthesia events (including surgery operations, the examination of MRI, day surgery, and invasive action). Subgroup analyses were conducted based on drug delivery methods, medication timing, DEX dosages, use of analgesics, event types, and recovery time.

**Results:**

A total of 33 trials involving 3395 patients were included. DEX significantly reduced the incidence of ED (odds ratios [OR] = 0.23, 95% confidence interval [CI]: 0.19–0.27, I^2^ = 37%, *P* < .00001). Intranasal delivery of DEX was the most effective (OR 0.18, 95% CI: 0.10–0.32, *P* < .00001, I^2^ = 0%). DEX also showed benefits in day surgery and mask insertion events (OR 0.30, 95% CI: 0.14–0.26, *P* = .001, I^2^ = 0%).

**Conclusion:**

DEX demonstrates superior efficacy in preventing ED in pediatric postanesthesia events compared to other sedatives and analgesics. Its use is recommended in various settings for its safety and effectiveness in managing ED.

## 1. Introduction

Delirium, a disorder of neurocognition characterized by alterations in attention and awareness^[1]^ is prevalent in pediatric patients, especially in hospitalized children, leading to serious outcomes.^[2]^ Emergence delirium (ED) is a form of psychomotor agitation and delirium that typically occurs about 45 minutes after anesthesia.^[3]^ Given the unique characteristics of children, the use of anesthetics is necessary during certain events in pediatric diagnosis and treatment, resulting in a high incidence of ED ranging from 10% to 80%.^[4]^ ED can lead to negative effects such as self-injury, prolonged recovery time, and dissatisfaction among parents and caregivers.^[5]^

Dexmedetomidine (DEX), a selective α-2 agonist, has been shown to prevent ED by exhibiting anxiolytic, analgesic, and sedative properties.^[6]^ Despite not being officially approved for pediatric use in any guidelines, DEX is widely used to prevent ED in various pediatric events.^[7]^ Compared to other sedative drugs, DEX has less impact on the nervous system, reduces the need for opioids and anesthetics,^[6]^ and is popular for managing postoperative complications in pediatric departments.

However, the application of DEX is limited in nonsurgical anesthesia procedures in pediatric diagnoses and treatments, such as day case surgeries, MRI examinations, and invasive catheterizations, due to factors like dosage, timing, and effectiveness. This systematic review and meta-analysis aim to evaluate the impact of DEX as an anesthesia adjuvant on ED in pediatric patients and to discuss any potential limitations.

## 2. Materials and methods

We searched the databases including “Pubmed,” “Web of Science,” and “EBSCO” of all resources through the PICOS (Population, Intervention, Comparison, Outcome, Study design) method until 13th October 2023. The entry words included “child” OR “children” OR “pediatric” AND “dexmedetomidine” OR “precedex” OR “MPV-1440” OR “MPV 1440” OR “Dexmedetomidine Hydrochloride” OR “Hydrochloride, Dexmedetomidine” AND “delirium” OR “Subacute Delirium” OR” Delirium, Subacute” OR” Deliriums, Subacute” OR” Subacute Deliriums” OR” Delirium of Mixed Origin” OR” Mixed Origin Delirium” OR” Mixed Origin Deliriums” AND “trails” and the search scope was “all fields.” Because all studies about the effect of DEX versus other drugs (placebo or other sedatives) on delirium in pediatric patients were eligible in this meta-analysis, we did not confine the search words of control drugs and study design, even non-drug. The inclusion criteria included the following: (1) participants with age <18 years; (2) management with prophylactic or therapeutic DEX and placebo or other sedatives or non-drug; (3) well-defined indicators associated with ED or comparisons of potential symptoms associated with the use of DEX; (4) randomized controlled trial. The exclusion criteria included the following: (1) participants with age ≥18 years; (2) management with DEX alone; (3) review or meta-analysis; (4) retrospective articles; (5) basic research; (6) article published as an abstract, letter, case report, editorial, note, method, or protocol; (7) article presented in non-English language.

Agitation is an unpleasant state of extreme arousal while delirium is a disturbance of consciousness and cognition.^[8]^ After discussion, we finally quit these articles because the conceptions of agitation and delirium are different. However, we preserve some articles of agitation for using Pediatric Anesthesia Emergence Delirium (PAED) scale to assess the eventual outcomes, because to date, the only validated scale is the PAED.^[5]^

Besides, some articles have no certain conclusion or the opposite result of anticipation about the effect of DEX and other sedatives. We also accept them aiming to analyze the possible causes of non-results for more comprehensive demonstration of DEX.

It is also important to note that due to the limited number of relevant surgical articles that met our criteria, the meta-analysis primarily focuses on the discussion of dexmedetomidine in preventing emergence delirium during short-term anesthesia in pediatric patients.

### 2.1. Data analysis

This meta-analysis aimed to analyze whether DEX had an advantage in reducing the incidence of ED of post-event in pediatric patients when compared with placebo or other sedatives or non-drug and the lack it may exist.

Two authors were independently responsible for reviewing the titles, abstracts, or both and summarized the data of the included literature. Another author was in charge of the data discrepancy adjustment.

Two authors were responsible for extracting the following information: (1) authors; (2) publication year; (3) number of the total participants in each study; (4) age range of all the participants; (5) country of publication; (6) procedures that the participants underwent; (7) time of DEX or other comparators administration; (8) types of other comparators; (9) infusion speed or dosage of DEX or other sedatives; (10) ways of drug delivery; (11) number of patients with ED following sedation or general anesthesia; and (12) other anesthetic medicines that may affect the results.

Two authors independently assessed the quality of the included trials. The risk of bias of randomized controlled trials (RCTs) was assessed by the Cochrane Collaboration Risk of Bias Assessment tool including 7 items: random sequence generation, allocation concealment, blinding of participants and personnel, blinding of outcome assessment, incomplete outcome data, selective reporting, and others (bias due to vested financial interest and academic bias). If a trial had one or more of the items to be judged as high or unclear risk of bias, this trial was classified as having high risk.^[9]^ If the 2 authors had different assessment results, they will be consulted by the third or the fourth one. Eventually, the authors reached a consensus. All included trials were grouped based on different control drugs.

RevMan Review Manager version 5.4 (Cochrane Collaboration, Oxford, UK) was used to perform statistical analyses. The values of I^2^ and the Mantel–Haenszel chi square test (*P*-value for heterogeneity) were used to evaluate the heterogeneity of included studies. And the values of I^2^ < 40%, 40 to 60%, and >60% represented low, moderate, and high heterogeneity, respectively.^[10]^ If I^2^ > 50% or a *P*-value for heterogeneity <0.1 was identified, the method of random-effect model analysis was applied to analyze the data. Conversely, if I^2^ < 50% or a *P*-value for heterogeneity .1 was presented, the method of a fixed-effect model was used.^[11]^ The dichotomous outcome was reported as odds ratios (OR) with 95% confidence interval (CI). The statistical tests were two-sided, and a *P*-value for overall effect <.05 was considered to have a significant difference.

Sensitivity analysis was conducted to solve the problem of significant heterogeneity (I^2^ > 20%) through the method of subgroup analysis or one-by-one literature removal. Meta-regression was used to investigate the heterogeneity sources for the group with I^2^ > 20% according to possible risk factors. A subgroup analysis proceeded based on the risk factor with *P* < .05 by meta-regression analysis; conversely, the method of one-by-one literature removal was used if *P*-values of all risk factors were .05 or more.

## 3. Results

### 3.1. Study collection and selection

**Figure 1.**
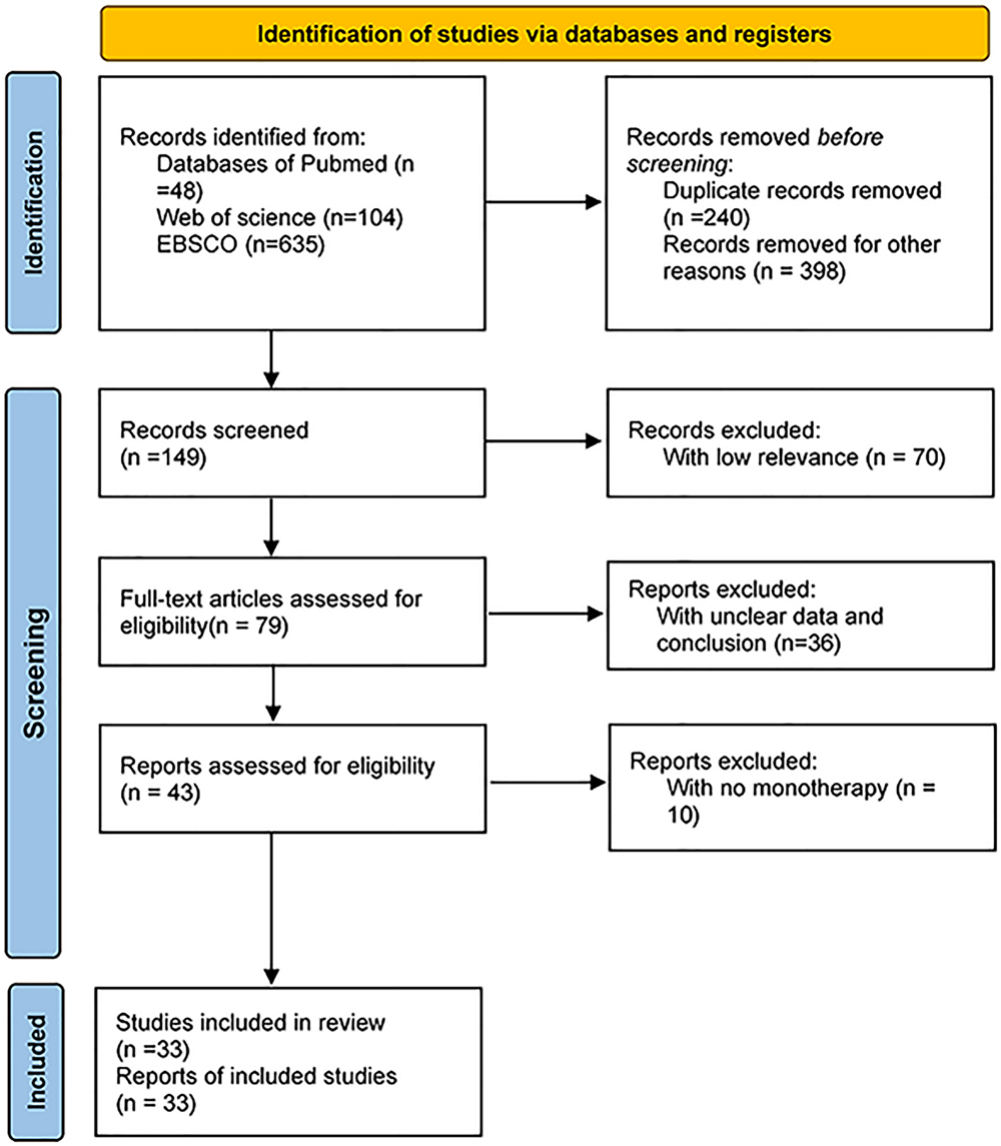
PRISMA flow diagram.

The screening process of the eligible literature is shown in Figure 1. We searched 48 trials from Pubmed, 635 from EBSCO, and 104 from Web of Science according to the inclusion criteria. Six hundred thirty eight trials were removed due to duplicates. Seventy trials were excluded because they did not meet the standard of inclusion criteria by scanning the titles and abstracts, and 36 trials were removed by scanning the full text. Eventually, 33 trials^[12–44]^ including 3395 patients were identified through our search strategy. We divided all included trials into several groups like different doses of DEX groups,^[12–17]^ saline groups,^[18–20,23,25–37,41–43]^ midazolam groups,^[21,22,24,36,38,39,44]^ propofol groups,^[18–20,40]^ ketamine,^[23]^ clonidine,^[39]^ esketamine,^[32]^ dezocine,^[29]^ ondansetron,^[31]^ dexamethasone^[31]^, and non-drug.^[36,37]^ We chose the different doses of DEX as a single group even though there are comparisons between DEX and placebo, but we wanted these groups to clarify the effect between various doses of DEX to find the safe dose to meet the needs of the clinic.

### 3.2. Characteristics of included trials

We chose 33 trials eventually, with 6 trials^[12–18]^ of 76 patients in the different doses of DEX groups, 19 trials^[18–20,23,25–31,33–37,41–43]^ of 1675 patients in the saline groups, 7 trials^[21,22,24,36,38,39,44]^ of 421 patients in the midazolam groups, 4 trails^[18–20,40]^ of 258 patients in the propofol groups, 5 trails^[23,29,31,32,39]^ of 358 patients in the ketamine, clonidine, esketamine, dezocine, ondansetron, dexamethasone groups respectively and 2 trails^[36,37]^ of 120 patients in the non-drug groups.

Table 1 summarizes the basic information of all included articles. These basic information included the title, the author, the region, the study methods, the type of event, the age of participants, dosage, the timing of drug administration, the comparator groups, the other anesthetic, and the routes of drug delivery. The summary of GRADE assessment of quality of evidence are shown in Table 2. All trails were prospective. Among these trials, there were 3 trials^[20,26,32]^ that described the influence during the examination of MRI, 1 trial^[38]^ described the postoperation in ICU, 1 trial^[16]^ was about mask placement, 1 trial^[17]^ was day case surgery, and the rest^[12–15,17–19,21–25,27–31,33–37,39–44]^ were about various types of surgery. Besides, otolaryngology surgeries include 10 trials.^[14,22,25,27–29,35,39,43]^ Dental surgeries or cleft palate surgery included 4 trials.^[18,21,24,31]^ Ophthalmic surgeries were including 8 trials.^[12,13,23,30,33,36,41,44]^ General or urological surgeries included 4 trials.^[15,19,37,40]^ Cardiac surgery included 1 trial.^[34]^ The ways of drug delivery were also different: the way of oral was related to 2 trials,^[24,39]^ the way of intranasal was related to 5 trials,^[14,16,21,36,44]^ 1 trial was about the comparison of the effect of intranasal and injection^[17]^, and the rest was about the way of injection.^[12,13,15,18–20,22,23,25–35,37,38,40–43]^ Also, the medication time was not the same. The trials of the drugs used before the event were 11.^[14,16,20,21,24,26,29,32,36,39,44]^ The trials of using after the event were 2.^[38,40]^ One trial was about the comparison of the effect of drugs were used before or during the event.^[17]^ And the rest were used during the event.^[12,13,15,18,19,22,23,25,27,28,30,31,33–35,37,41–43]^ The number of patients with ED in DEX and comparator groups are also collected (see Table S1, Supplemental Digital Content, http://links.lww.com/MD/N385 which illustrates the number of patients with ED in DEX and comparator groups and Table S2, Supplemental Digital Content, http://links.lww.com/MD/N386 which illustrates the number of patients with ED in different doses of DEX and comparator groups).

**Table 1:** The basic information of all included trials.

Study	N	Age (year)	Method	Event	Medication time	Dex dosage (μg/kg)	Comparator group	Other medicine
Aydogan 2013^[38]^	32	12–17	RCT	Postoperation in ICU	Postoperation	0.4/h	Midazolam	Fentanyl
Abdel-Rahman 2018^[13]^	90	3–8	RCT	Strabismus surgery	During	0.25, 0.5	Saline	Sevoflurane
Bong 2015^[20]^	120	2–7	RCT	MRI	Before	0.3	Propofol saline	Sevoflurane
Bromfalk 2023^[39]^	90	2–6	RCT	Ear, nose, and throat surgery	Before	2	Midazolam clonidine	Propofol remifentanil sevoflurane
Cao 2016^[35]^	60	2–8	RCT	Tonsillectomy surgery	During	0.5/h	Saline	Sevoflurane fentanyl etomidate propofol sufentanil
Chen 2013^[23]^	78	2–7	RCT	Strabismus surgery	During	1/h	Ketamine saline	Sevoflurane
Chen 2018^[15]^	100	3–7	RCT	Inguinal hernia repair surgery	During	0.25, 0.5, 0.75, 1	Saline	Propofol sevoflurane
Cho 2020^[22]^	66	2–12	RCT	Tonsillectomy surgery	During	0.3	Midazolam	Sevoflurane ketorolac
Elghamry 2021^[33]^	70	3–7	RCT	Strabismus surgery	During	0.3	Saline	Sevoflurane fentanyl ketorolac
Hadi 2015^[42]^	92	3–7	RCT	Adenotonsillectomy surgery	During	0.3	Saline	Sevoflurane fentanyl
Han 2022^[40]^	53	1–14	RCT	Surgery	After	0.5	Propofol	Sevoflurane fentanyl remifentanil pentazocine
Hauber 2015^[25]^	393	4–10	RCT	Tonsillectomy surgery	During	0.5	Saline	Propofol sevoflurane morphine
Huang 2022^[18]^	86	0.6–2	RCT	Cleft palate Surgery	During	0.5/h	Propofol saline	Remifentanil sevoflurane sufentanil
Isik 2006^[26]^	42	1.5–10	RCT	MRI	Before	1	Saline	Sevoflurane
Jia 2017^[29]^	93	7–18	RCT	Tonsillectomy surgery	Before	1	Dezocine saline	Propofol fentanyl sevoflurane
Lee-Archer 2020^[17]^	234	2–7	RCT	Day case surgery	Before during	2, 1	Saline	Inhaled or intravenous general anesthetics (not specifically mentioned)
Li 2018^[14]^	90	2–7	RCT	Adenoidectomy surgery	Before	1, 2	Saline	Remifentanil propofol ketamine
Makkar 2016^[19]^	100	2–8	RCT	Infraumbilical surgery	During	0.3	Propofol saline	Desflurane sevoflurane
Mountain 2011^[24]^	41	1–6	RCT	Dental restoration	Before	4	Midazolam	Sevoflurane fentanyl isoflurane
Pandey 2022^[30]^	152	1–6	RCT	Ophthalmic surgery	During	0.3	Saline	Midazolam desflurane sevoflurane propofol fentanyl
Raman 2023^[41]^	101	2–12	RCT	Ophthalmic surgery	During	0.4	Saline	Propofol fentanyl sevoflurane
Ramlan 2021^[44]^	64	1–12	RCT	Ophthalmic surgery	Before	1	Midazolam	Sevoflurane
Shama 2023^[31]^	100	6–12	RCT	Dental rehabilitation surgery	During	0.3	Dexamethasone ondansetron saline	Midazolam sevoflurane fentanyl
Shi 2019^[27]^	90	2–7	RCT	Tonsillectomy surgery	During	0.5	Saline	Propofol fentanyl sevoflurane
Soliman 2022^[37]^	90	4–9	RCT	Hypospadias repair surgery	During	0.2	Saline records from mum	Propofol fentanyl sevoflurane
Song 2016^[12]^	103	2–6	RCT	Strabismus surgery	During	0.25, 0.5, 1	Saline	Desflurane sevoflurane
Sun 2017^[34]^	50	1–6	RCT	Cardiac surgery	During	0.5/h	Saline	Etomidate midazolam sufentanil sevoflurane
Tsiotou 2018^[28]^	60	3–14	RCT	Tonsillectomy surgery	During	1	Saline	Propofol fentanyl midazolam remifentanil nalbuphine
Wang 2020^[21]^	60	3–6	RCT	Dental surgery	Before	2	Midazolam	Sevoflurane fentanyl remifentanil sufentanil
Xu 2022^[32]^	111	0.5–8	RCT	MRI	Before	0.3	Esketamine	Propofol
Yao 2015^[16]^	89	3–7	RCT	Mask insertion	Before	1, 2	Saline	Propofol sevoflurane
Yao 2020^[36]^	153	2–6	RCT	Strabismus surgery	Before	2	Midazolam saline	Propofol sevoflurane sufentanil
Zhang 2022^[43]^	80	3–7	RCT	Tonsillectomy surgery	During	0.4	Saline	Propofol sevoflurane tramadol

RCT: randomized controlled trial.

**Table 2:** Summary of findings table with GRADE assessment quality of evidence.

Certainty assessment							No. of patients		Effect		Certainty	Importance
No. of studies	Study design	Risk of bias	Inconsistency	Indirectness	Imprecision	Other considerations	Comparison of pediatric ED between dexmedetomidine	Saline groups	Relative (95% CI)	Absolute (95% CI)		
Comparison of pediatric ED between dexmedetomidine and saline groups												
25	Randomized trials	Not serious	Not serious	Not serious	Not serious	None	191/1264 (15.1%)	550/1251 (44.0%)	OR 0.19 (0.15–0.23)	310 fewer per 1000 (from 334 fewer to 287 fewer)	⨁⨁⨁⨁ High	IMPORTANT
Comparison of pediatric ED between dexmedetomidine and midazolam groups												
7	Randomized trials	Not serious	Not serious	Not serious	Not serious	None	25/216 (11.6%)	65/205 (31.7%)	OR 0.28 (0.17–0.47)	202 fewer per 1000 (from 244 fewer to 138 fewer)	⨁⨁⨁⨁ High	IMPORTANT
Comparison of pediatric ED between dexmedetomidine and propofol groups												
3	Randomized trials	Not serious	Not serious	Not serious	Very serious*	Publication bias strongly suspected	9/87 (10.3%)	30/92 (32.6%)	OR 0.21 (0.09–0.50)	234 fewer per 1000 (from 284 fewer to 131 fewer)	⨁◯◯◯ Very low	NOT IMPORTANT
Comparison of pediatric ED between dexmedetomidine and other groups (including esketamine, dexamethasone, ondansetron, dezocine, and ketamine)												
7	Randomized trials	Not serious	Not serious	Not serious	Not serious	None	25/253 (9.9%)	46/250 (18.4%)	OR 0.44 (0.25–0.78)	94 fewer per 1000 (from 131 fewer to 34 fewer)	⨁⨁⨁⨁ High	IMPORTANT
Comparison of pediatric ED between dexmedetomidine and all comparator groups												
33	Randomized trials	Not serious	Not serious	Not serious	Not serious	None	282/1938 (14.6%)	736/1912 (38.5%)	OR 0.23 (0.19–0.27)	259 fewer per 1000 (from 279 fewer to 240 fewer)	⨁⨁⨁⨁ High	CRITICAL

* Fewer than 5 studies.

### 3.3. Bias risk assessment

**Figure 2.**
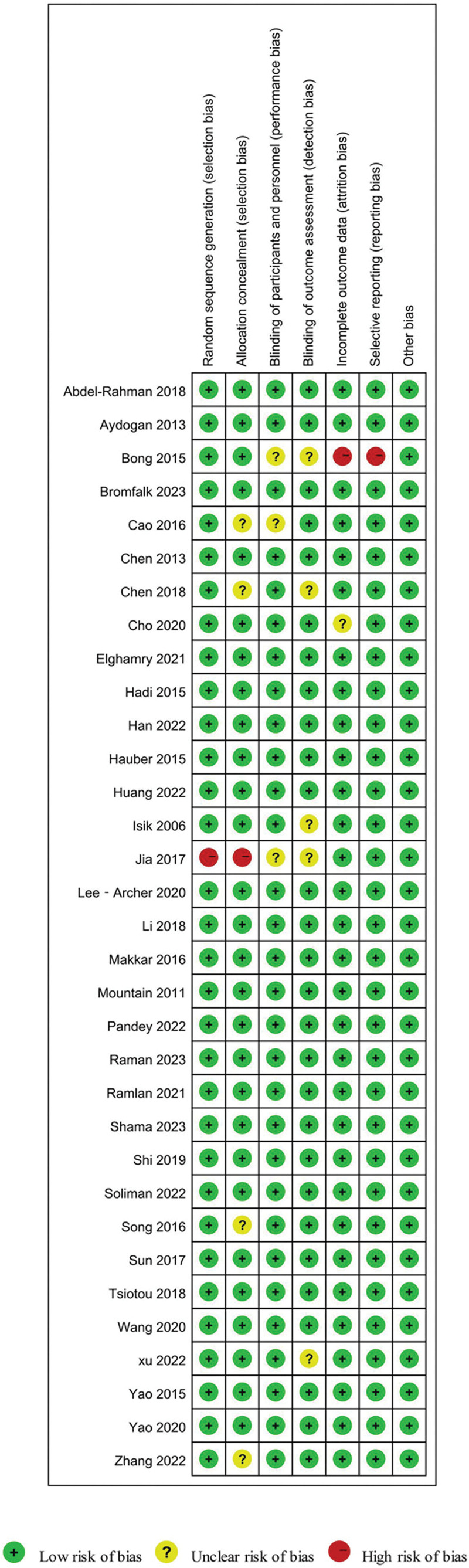
Risk of bias summary review authors’ judgments about each risk of bias item for each included study.

Bias risk of 33 RCTs was assessed by the Cochrane Collaboration Risk of Bias Assessment tool. Random sequence generation was assessed as a low risk of bias in 32 studies (97%), allocation concealment was assessed in 28 studies (85%), blinding of participants was assessed in 30 studies (91%), blinding of outcome assessment was assessed in 28 studies (85%), incomplete outcome data were assessed in 31 studies (94%), and selective outcome reporting was assessed in 32 studies (97%). Twenty four RCTs ^[13,14,16–19,21,23–25,27,28,30,31,33,34,36–42,44]^ were assessed to be of high quality (see Fig. 2 and Figure S1, Supplemental Digital Content, http://links.lww.com/MD/N387 which illustrates the risk of bias graph review authors judgments about each risk of bias item presented as percentages across all included studies).

### 3.4. Post-event incidence of ED

Firstly, we evaluated the effect of DEX administration on ED compared with all comparator groups including different dosages of DEX.^[12–44]^ Then we assessed saline (including various dosages of DEX),^[12–20,23,25–31,33–37,41–43]^ propofol,^[18–20,40]^ midazolam,^[21,22,24,36,38,39,44]^ and the rest.^[23,29,31,32,36,37,39]^ The standard of ED was those patients whose PAED score was over 10 (including severe ED whose PAED score was over 12 or 15).

The random-effect model with OR was chosen because of high I^2^ in propofol groups (I^2^ = 61%), whereas the fixed-effect model with OR was selected due to low I^2^ in all comparator groups (I^2^ = 37%), saline groups (I^2^ = 38%), midazolam groups (I^2^ = 20%), and other groups (I^2^ = 0%).

**Figure 3.**
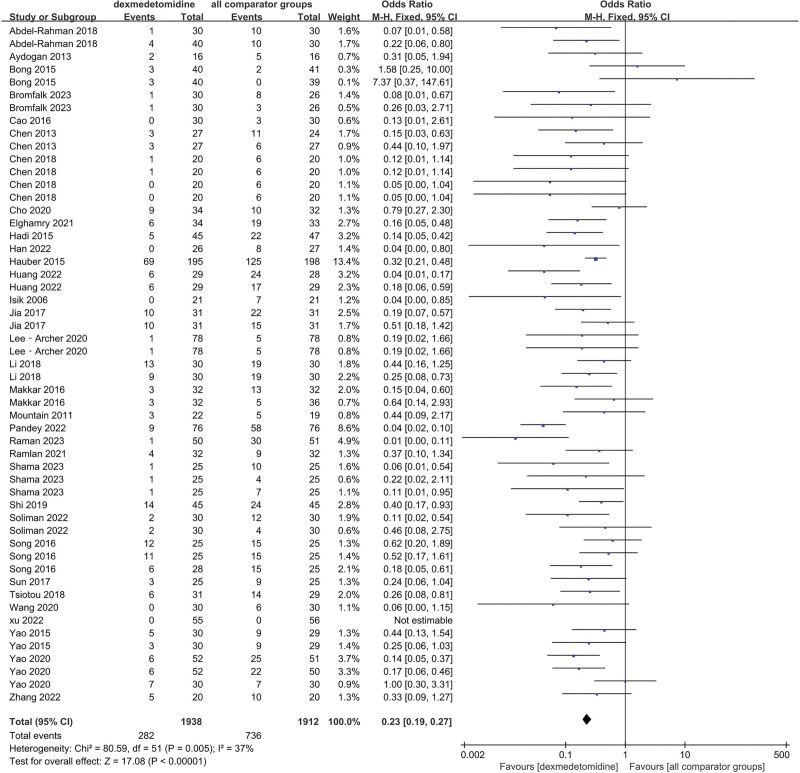
Comparison of pediatric ED between DEX and all comparator groups.

**Figure 4.**
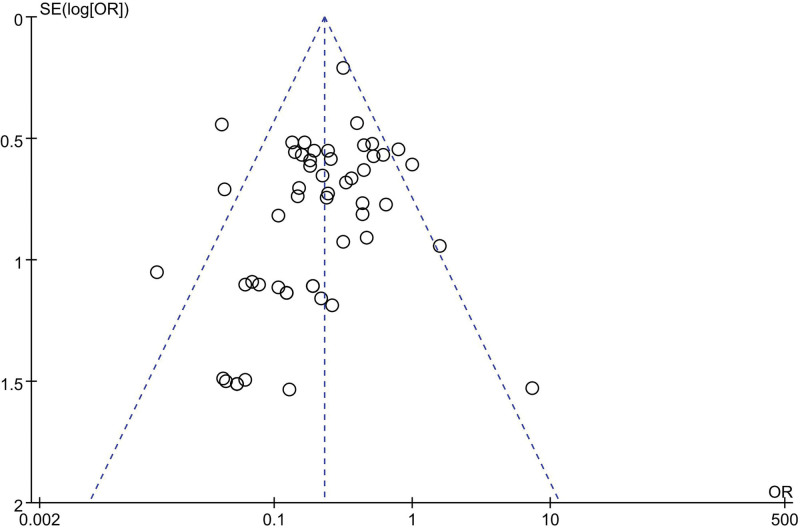
The funnel plot of comparison of pediatric ED between DEX and all comparator groups.

**Figure 5.**
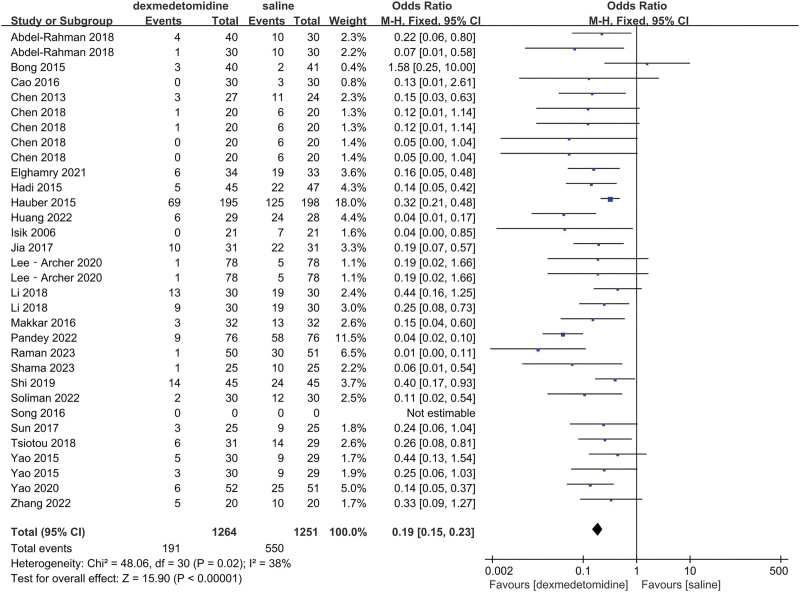
Comparison of pediatric ED between DEX and saline groups.

**Figure 6.**
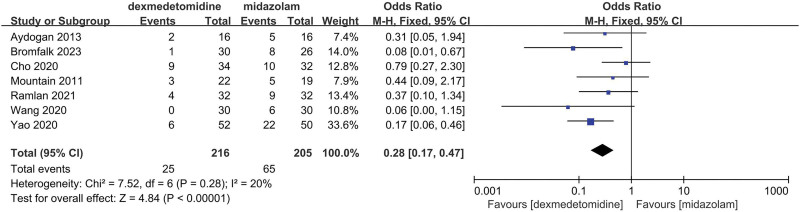
Comparison of pediatric ED between DEX and midazolam groups.

**Figure 7.**
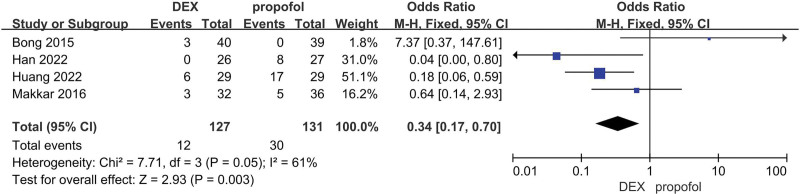
Comparison of pediatric ED between DEX and propofol groups.

**Figure 8.**
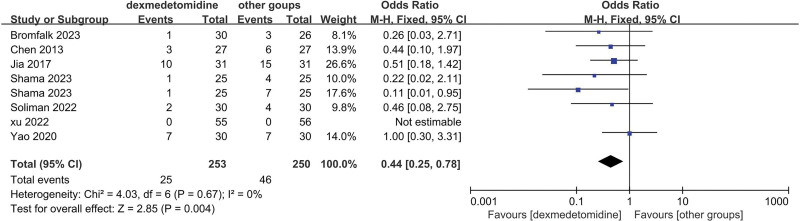
Comparison of pediatric ED between DEX and other groups.

The final results showed that there is a great difference in the incidence of ED after analyzing the data of DEX and all comparator groups (OR = 0.23, 95% CI: [0.19, 0.27], I^2^ = 37%, *P* for effect < .00001) (Figs. 3 and 4). Compared to saline groups, the result was (OR = 0.19, 95% CI: [0.15, 0.23], I^2^ = 38%, *P* for effect < .00001) (see Fig. 5 and Figure S2, Supplemental Digital Content, http://links.lww.com/MD/N387 which illustrates the funnel plot of comparison of pediatric ED between DEX and saline groups). Compared to midazolam groups, the result was (OR = 0.28, 95% CI: [0.17, 0.47], I^2^ = 20%, *P* for effect < .00001) (Fig. 6). Compared to propofol groups, the result was (OR = 0.39, 95% CI: [0.08, 1.84], I^2^ = 61%, *P* for effect < .00001) (Fig. 7). Compared to other groups, the result was (OR = 0.44, 95% CI: [0.25, 0.78], I^2^ = 0%, *P* for effect < .00001) (Fig. 8).

Incidentally, the number of articles of the drugs in other groups was only 1 or 2 literature with no heterogenicity if evaluating respectively. Comparing DEX with clonidine (OR = 0.26, 95% CI: [0.03, 2.71], *P* for effect = .26), dezocine (OR = 0.51, 95% CI: [0.18, 1.42], *P* for effect = .20), ondansetron (OR = 0.11, 95% CI: [0.01, 0.95], *P* for effect = .04), dexamethasone (OR = 0.22, 95% CI: [0.02, 2.11], *P* for effect = .19), ketamine (OR = 0.44, 95% CI: [0.10, 1.97], *P* for effect = .28], and non-drug (OR = 0.78, 95% CI: [0.29, 2.08], *P* for effect = .62), we found there is no significant difference. So we try to collect whole the other groups.

### 3.5. Sensitivity analysis

We used the method of one-by-one literature removal to investigate the heterogeneity sources. Four trials^[18,20,30,41]^ were found to be the main sources of heterogeneity in all comparator groups (I^2^ dropped from 37–0%), 3 trials^[20,30,41]^ in the saline group (I^2^ dropped from 38–0%), 1 trail^[22]^ in the midazolam groups (I^2^ dropped from 20–0%), and 2 trails^[19,20]^ in the propofol groups (I^2^ dropped from 61–0%).

The post results were performed by the fixed-effects model with OR, and the final results were consistent with those prior to the sensitivity analysis: all comparator groups: (OR = 0.26, 95% CI: [0.21, 0.32], I^2^ = 0%, *P* for effect < .00001) (see Figure S3, Supplemental Digital Content, http://links.lww.com/MD/N387 which illustrates the comparison of pediatric ED between DEX and all comparator groups after sensitivity analysis and Figure S4, Supplemental Digital Content, http://links.lww.com/MD/N387 which illustrates the funnel plot of comparison of pediatric ED between DEX and all comparator groups after sensitivity analysis); saline groups: (OR = 0.24, 95% CI: [0.18, 0.31], I^2^ = 0%, *P* for effect < .00001) (see Figure S5, Supplemental Digital Content, http://links.lww.com/MD/N387 which illustrates the comparison of pediatric ED between DEX and saline groups after sensitivity analysis and Figure S6, Supplemental Digital Content, http://links.lww.com/MD/N387 which illustrates the funnel plot of comparison of pediatric ED between DEX and saline groups after sensitivity analysis); midazolam groups: (OR = 0.21, 95% CI: [0.11, 0.38], I^2^ = 0%, *P* for effect < .00001) (see Figure S7, Supplemental Digital Content, http://links.lww.com/MD/N387 which illustrates the comparison of pediatric ED between DEX and midazolam groups after sensitivity analysis); propofol groups: (OR = 0.13, 95% CI: [0.05, 0.38], I^2^ = 0%, *P* for effect = .0002) (see Figure S8, Supplemental Digital Content, http://links.lww.com/MD/N387 which illustrates the comparison of pediatric ED between DEX and propofol groups after sensitivity analysis).

### 3.6. Subgroups analysis

To further search the effects of DEX on ED incidence, we conducted the subgroup analysis in 5 parts: ways of drug delivery (oral, intravenous, and intranasal), medication time (premedication, during the event, and before the event finishing), the different dosages of DEX (low dose [<0.5 μg/kg], moderate dose [0.5–1 μg/kg], high dose [>1 μg/kg], continuous dosage, and both of bolus and continuous dosage), with or not analgesic (with one analgesic, with 2 analgesics, with 3 analgesics, and with no analgesic), and type of event (surgery, MRI, and other events). The outcomes of subgroup analysis are displayed in Table 3.

**Table 3:** The outcomes of subgroup analysis and recovery time between different dosages of DEX after events.

Outcomes/subgroup	No. of studies	No. of patients	Heterogeneity	Model of pool	Effect size (95% CI:)		*P*-value			Subgroup difference
Ways of drug delivery										*P* = .93
Intravenous	26	4970	I^2^ = 45%	Fixed effect	OR 0.22 [0.18, 0.26]		*P* < .00001			
Intranasal	6	613	I^2^ = 0%	Fixed effect	OR 0.24 [0.16, 0.36]		*P* < .00001			
Oral	2	82	I^2^ = 0%	Fixed effect	OR 0.22 [0.08, 0.64]		*P* = .005			
Medication time										*P* = .49
Before	12	1102	I^2^ = 13%	Random effect	OR 0.28 [0.20, 0.41]		*P* < .00001			
During	15	1266	I^2^ = 14%	Random effect	OR 0.19 [0.14, 0.27]		*P* < .00001			
Before event finishing	5	778	I^2^ = 80%	Random effect	OR 0.23 [0.10, 0.53]		*P* = .0007			
After	2	85	I^2^ = 28%	Random effect	OR 0.16 [0.02, 1.09]		*P* = .06			
DEX dosages										*P* = .10
Low dose (<0.5 μg/kg)	8	562	I^2^ = 44%	Fixed effect	OR 0.16 [0.10, 0.26]		*P* < .00001			
Moderate dose (0.5–1 μg/kg)	13	1288	I^2^ = 0%	Fixed effect	OR 0.28 [0.22, 0.36]		*P* < .00001			
High dose (>1 μg/kg)	7	612	I^2^ = 0%	Fixed effect	OR 0.18 [0.11, 0.29]		*P* < .00001			
Continuous dosage	4	228	I^2^ = 17%	Fixed effect	OR 0.13 [0.06, 0.27]		*P* < .00001			
Both	1	78	I^2^ = 4%	Fixed effect	OR 0.25 [0.09, 0.69]		*P* = .007			
With or not analgesic										*P* = .15
With 1 analgesic	15	1546	I^2^ = 52%	Random effect	OR 0.23 [0.16, 0.33]		*P* < .00001			
With 2 analgesics	3	213	I^2^ = 0%	Random effect	OR 0.12 [0.06, 0.24]		*P* < .00001			
With 3 analgesics	3	173	I^2^ = 0%	Random effect	OR 0.18 [0.06, 0.48]		*P* = .0007			
With no analgesic	10	897	I^2^ = 18%	Random effect	OR 0.30 [0.20, 0.44]		*P* < .00001			
Type of event										*P* = .47
Surgery	27	2624	I2 = 38%	Random effect	OR 0.22 [0.17, 0.28]		*P* < .00001			
MRI	3	273	I2 = 69%	Random effect	OR 0.85 [0.06, 12.23]		*P* = .90			
Other events	3	355	I2 = 0%	Random effect	OR 0.30 [0.14, 0.62]		*P* = .001			
Recovery time (min)									*P* = .0006	
Low dose of DEX (<0.5 μg/kg)	5	410	I^2^ = 76%	Random effect		MD 2.92 [1.33, 4.51]		*P* = .0003		
High dose of DEX (0.5–1 μg/kg)	5	653	I^2^ = 96%	Random effect		MD13.54 [7.39, 19.68]		*P* < .0001		
DEX 0.5 μg/kg/h	2	146	I^2^ = 21%	Random effect		MD1.51 [0.26, 2.76]		*P* = .02		

### 3.7. Way of drug delivery

Our research discussed that DEX effectively decreased the incidence of ED in the intravenous way (OR 0.21, 95% CI: 0.14–0.30, *P* < .00001, I^2^ = 52%), intranasal way (OR 0.18, 95% CI: 0.10–0.32, *P* < .00001, I^2^ = 0%), and oral way (OR 0.25, 95% CI: 0.08–0.75, *P* = .01, I^2^ = 0%). The most effective route may be the intranasal way (see Figure S9, Supplemental Digital Content, http://links.lww.com/MD/N387 which illustrates the comparison of pediatric ED between dexmedetomidine and all comparator groups [subgroup of different ways of drug delivery]). This way is simple to act and suitable for those children who are out of control.

### 3.8. Medication time

When adding the subgroup of medication time, we found except post-event (OR 0.16, 95% CI: 0.02–1.09, *P* = .06, I^2^ = 28%) has no significant difference. The other groups (before the event [OR 0.28, 95% CI: 0.20–0.41, *P* < .00001, I^2^ = 13%], during the event [OR 0.19, 95% CI: 0.14–0.27, *P* < .00001, I^2^ = 14%], and before the event finishing [OR 0.23, 95% CI: 0.10–0.53, *P* = .0007, I^2^ = 80%]) could effectively decrease the incidence of ED. When administrating DEX during the processes especially after induction of anesthesia would acquire the best result (see Figure S10, Supplemental Digital Content, http://links.lww.com/MD/N387 which illustrates the comparison of pediatric ED between dexmedetomidine and all comparator groups [subgroup of different medication time]).

### 3.9. Dosages of DEX

Then we conducted another subgroup of various dose of DEX, all types (low dose [<0.5 μg/kg] [OR 0.16, 95% CI: 0.10–0.26, *P* < .00001, I^2^ = 44%], moderate dose [0.5–1 μg/kg] [OR 0.28, 95% CI: 0.22–0.36, *P* < .00001, I^2^ = 0%], high dose [>1 μg/kg] [OR 0.18, 95% CI: 0.11–0.29, *P* < .00001, I2 = 0%], continuous dosage [OR 0.13, 95% CI: 0.06–0.27, *P* < .00001, I^2^ = 17%], and both of bolus and continuous dosage [OR 0.25, 95% CI: 0.09–0.69, *P* < .00001, I^2^ = 5%]) could reduce the incidence of ED. Compared to the single bolus dose, a continuous dosage would be better (see Figure S11, Supplemental Digital Content, http://links.lww.com/MD/N387 which illustrates the comparison of pediatric ED between dexmedetomidine and all comparator groups [subgroup of different DEX dosages]).

### 3.10. With or not analgesic

Further subgroup analysis was performed as the situation of analgesic. The results were that all groups (with 1 analgesic [OR 0.23, 95% CI: 0.16–0.33, *P* < .00001, I^2^ = 52%], with 2 analgesics [OR 0.12, 95% CI: 0.06–0.24, *P* < .00001, I^2^ = 0%], with 3 analgesics [OR 0.18, 95% CI: 0.06–0.48, *P* = .0007, I^2^ = 0%], and with no analgesic [OR 0.30, 95% CI: 0.20–0.44, *P* = .02, I^2^ = 18%]) could decrease the incidence of ED, and with 2 analgesics got the better result (see Figure S12, Supplemental Digital Content, http://links.lww.com/MD/N387 which illustrates the comparison of pediatric ED between dexmedetomidine and all comparator groups [subgroup of whether analgesic]).

### 3.11. Type of event

Another subgroup analysis was according to the type of event. As follows were the results: the event of surgery (OR 0.22, 95% CI: 0.17–0.28, *P* < .00001, I^2^ = 38%), the event of MRI (OR 0.85, 95% CI: 0.06–12.23, *P* = .90, I^2^ = 69%), and other event (OR 0.30, 95% CI: 0.14–0.26, *P* = .001, I^2^ = 0%). There was no difference in the event of MRI. The effect of DEX was better in the event of surgery than in other events (see Figure S13, Supplemental Digital Content, http://links.lww.com/MD/N387 which illustrates the comparison of pediatric ED between dexmedetomidine and all comparator groups [subgroup of different events]).

### 3.12. Secondary outcomes

For investigating the comprehensive effects of DEX on patients in pediatrics, we tried to find the differences between DEX and other comparator groups on sedation effect before the events and the differences in recovery time after the events. Unfortunately, we could not explore any evidence or data about the effects before the events, but we found some data covering the recovery time after the events finished. And the outcomes are shown in Table 3.

Compared to other comparator groups, we found that DEX delayed the recovery time of children. And different dosages had different effect (low dose of DEX [<0.5 μg/kg] [mean difference [MD] 2.92, 95% CI: 1.33–4.51, *P* = .0003, I^2^ = 76%], high dose of DEX [0.5–1 μg/kg] [MD 13.54, 95% CI: 7.39–19.68, *P* < .0001, I^2^ = 96%], and continuous dosage [0.5 μg/kg/h] [MD 1.48, 95% CI: 0.37–2.59, *P* = .009, I^2^ = 21%] *P*-value = .0006). The more doses used, the more time recovering (the recovery time prolonged as the dose of DEX increased. Continuous dosage could reduce the recovery time to a certain degree [see Figure S14, Supplemental Digital Content, http://links.lww.com/MD/N387 which illustrates the comparison of pediatric ED between dexmedetomidine and all comparator groups [subgroup of recovery time]]).

## 4. Discussion

The results of this meta-analysis suggested that DEX has more significant advantages in preventing ED in pediatric patients undergoing anesthesia compared to other methods, particularly in surgical settings. In the past, short-acting sedatives such as propofol, midazolam, and ketamine were commonly used in pediatric clinics. Ketamine, an N-methyl D-aspartate antagonist, exerts sedative effects by inhibiting glutamate from binding to the N-methyl D-aspartate receptor. Because ketamine interacts with central nervous system receptors, including μ, κ, and δ opioid receptors, it also possesses analgesic properties.^[45]^ However, ketamine can lead to postoperative psychomimetic effects like ED, which are dose-dependent. On the other hand, alpha-2 adrenoceptor agonists like DEX can prevent these reactions.^[46]^ Midazolam, a benzodiazepine, acts as a gamma-aminobutyric acid agonist and is widely used for its anxiolytic effects^[47]^ but has been associated with delirium at higher doses.^[48]^ Propofol functions by enhancing the neuro-inhibitory activity of gamma-aminobutyric acid. Its rapid onset of action and short half-life facilitate a faster awakening once the infusion is discontinued. Unfortunately, propofol is associated with several unavoidable adverse effects. The most common is pain at the injection site. Additionally, airway obstruction and apnea are significant concerns that cannot be ignored. Other adverse effects include bradycardia, hypotension, and central nervous system excitation, which can potentially trigger seizures, among others.^[49]^ Moreover, neither midazolam nor propofol provides analgesic effects.

Our data clearly demonstrated that DEX has significant benefits in preventing ED, a common and challenging complication in pediatric medical procedures.^[3]^ ED not only increases the likelihood of additional complications, such as self-injury and prolonged recovery times but also impacts the satisfaction levels of both parents and caregivers.^[9]^ Numerous factors contribute to the onset of ED, including the use of fast-acting volatile anesthetics, patient age, gender, surgical stimuli, postoperative pain, and anxiety due to separation from parents.^[50]^ Primarily, there is an increase in cytokines, notably IL-6 and TNF-alpha, which lead to the development of neuroinflammation.^[51]^ Furthermore, delirium, which disrupts the function of higher cortical centers, is associated with postoperative anxiety and sleep disturbances that can persist for up to 14 days, potentially leading to long-term sequelae.^[52–54]^ DEX is particularly effective because it readily crosses the blood–brain barrier^[55]^ and has been proven to reduce IL-6 cytokine levels in patients who undergo total intravenous anesthesia.^[56]^ Additionally, DEX has been shown to lower levels of cortisol, C-reactive protein, TNF-alpha, and other interleukins such as IL-1β both 1 hour and 24 hours post-surgery.^[57]^ Moreover, DEX enhances neuroinflammatory behavior by suppressing inflammatory mediators, controlling apoptotic signaling pathways, and reducing the generation of oxygen-free radicals.^[58]^ In conclusion, DEX plays a crucial role in protecting the brain during episodes of delirium, highlighting its importance in pediatric anesthetic practice.

As previously noted, postoperative pain is a significant factor contributing to ED. DEX serves not only as an analgesic but also reduces the required dosage of opioids. Studies indicate that DEX lessens perioperative pain and the need for analgesics, while significantly extending the duration of analgesia during the first 24 hours.^[7]^ Aydogan, in his study, compared opioid dosages between patients administered DEX and those given midazolam.^[38]^ The findings revealed that the DEX group required a lower dose of opioids than the midazolam group. Consequently, DEX can decrease both the risk and potential addiction associated with opioid use. This is particularly important considering that children, especially neonates, are believed to have immature metabolic systems.^[59]^ DEX is advantageous in terms of safety due to its short elimination half-life; it is rapidly transformed into inactive metabolites through hepatic biotransformation.^[7]^ Furthermore, DEX minimally impacts the respiratory system and has been shown to reduce the incidence of postoperative nausea and vomiting.^[7]^ However, it is not without side effects, which include hypertension, hypotension, and bradycardia, among others.^[6]^ All the studies we reviewed acknowledged these issues but indicated that these symptoms are manageable and do not necessitate the use of any antagonists.

Although some literature indicated that DEX was less effective than propofol in preventing ED during MRI examinations, this is due to the use of suboptimal doses of DEX. Such literatures were excluded during sensitivity analysis. For example, Fang stated in his meta-analysis that propofol is preferable to DEX for MRI procedures, citing better outcomes in terms of recovery time and prevention of ED.^[60]^ Bong noted in his study that not only did the propofol group outperform the DEX group, but even the saline group achieved more favorable results than those receiving DEX. It is important to note that in their researches, the dosage of DEX was 0.3 μg/kg, administered without any sedatives other than sevoflurane,^[20]^ which has been linked to an increased risk of ED.^[3]^ In Xu’s article, a comparison was made between DEX and ketamine, both administered alongside a continuous infusion of propofol. The conclusion drawn was that ketamine and DEX had similar effects in preventing ED. However, due to the sedative effects of propofol, it is challenging to effectively distinguish between the impacts of ketamine and DEX on preventing ED. Moreover, the dosage of DEX was 0.3 μg/kg, which is considerably insufficient without other sedatives.^[32]^ Considering the slower onset of action of DEX,^[61]^ with a dose of 1 μg/kg taking effect after 45 minutes,^[55]^ a loading dose is generally administered before events when using DEX. In children and adolescents, the recommended loading doses range from 0.5 to 2 μg/kg administered over 10 minutes, followed by a recommended maintenance dose of 0.5 to 1 μg/kg/h. The dosage should be reduced when used in preterm neonates or neonates.^[62]^

In our meta-analysis, a low dose (<0.5 μg/kg) emerged as the better choice for preventing ED when administered in a single bolus. However, the most effective method appeared to be the use of a continuous dose throughout the procedure. Nonetheless, it is important to note that in the reviewed articles, there was always the concurrent use of at least 1 sedative or analgesic alongside DEX. These sedatives and analgesics typically have a synergistic effect with DEX, which can lead to an unclear understanding of a safe dose. Although Manning suggests in his article that 0.5 μg/kg may be the appropriate dose to prevent ED and that exceeding this dose does not provide additional protection,^[50]^ Mantecón-Fernández indicates that the effective continuous dose ranges from 0.1 to 2 μg/kg/h.^[59]^ However, the dosages of other sedatives used concurrently were not discussed in these studies. Therefore, given the existing uncertainties in diagnosis and treatment, ongoing research is essential to determine the optimal dosages of DEX and other medications when used simultaneously.

Regarding recovery time, when the dosage exceeds 3 μg/kg, the recovery time does not always increase with the dosage; instead, it remains between 44 and 91 minutes.^[61]^ Sin and colleagues assessed the impact of DEX on Postanesthesia Care Unit stay duration in their meta-analysis, revealing that the stay duration for the DEX group was only 0.69 (MD) minutes longer compared to the placebo group. Therefore, DEX does not delay recovery time.^[63]^

Considering the complexities of treating pediatric patients, we compared different routes of administration and found that the intranasal route yielded the best outcomes. This method delivers the drug directly to the mucosa, thereby avoiding hepatic first-pass metabolism and theoretically achieving plasma levels equivalent to those of an intravenous dose.^[64]^ This approach is particularly suitable for patients who experience pre-event anxiety, separation anxiety from their parents, and exhibit a lack of cooperation. It is favored because it has no irritating odor, does not cause respiratory depression, and is easier to administer than oral and intravenous routes^.[65,66]^ Apart from the traditional methods, the drug can also be administered using an atomizer. This method shows no significant difference in bioavailability, median onset time, and duration of sedation compared to the intranasal route and is easier for patients to accept.^[61]^ In addition to the routes of delivery discussed previously, there are numerous other methods such as intramuscular, epidural, intrathecal, interfascial, perineural, and even peritonsillar routes.^[7]^ These are predominantly used during pediatric surgeries to reduce perioperative pain. However, we have not found articles linking these routes to delirium, and therefore, they are not discussed in this meta-analysis.

### 4.1. Shortcomings

Our meta-analysis clearly delineates between the concepts of delirium and agitation, though it does include certain studies that assess agitation using the PAED scale. Consequently, the precision of the data might be somewhat compromised. Additionally, in our comparison groups, we found only 1 article each for interventions such as chloral hydrate, melatonin, xenon, and physical methods. A similar scarcity of literature was noted for clonidine, dezocine, ondansetron, dexamethasone, ketamine, and esketamine. Due to various factors, these data sets demonstrated either no significant differences or lacked representativeness when compared with the DEX groups. As a result, these medications were not included in our meta-analysis. Moreover, our study is subject to several other limitations, including variability in population characteristics, small sample sizes within each subgroup, inconsistent dosages and regimens of DEX, diverse types of concomitant medications, and differences in primary outcomes. Regrettably, these constraints prevented a more detailed stratification of the articles in our analysis.

## 5. Conclusion

DEX offers significant advantages in preventing ED in pediatric anesthesia, especially in short-term anesthesia events. As an anesthesia adjuvant, it has minimal impact on the respiratory and cardiovascular systems, provides potential brain protection, and results in low rates of postoperative nausea and vomiting. Its various delivery methods make it suitable for use in busy pediatric settings. However, careful patient selection and dosage are necessary since DEX is used “off-label” in children. While there is potential for DEX to replace certain hypnotics, further research is needed to fully understand its benefits and limitations in pediatric care.

## Acknowledgments

This review was registered in PROSPERO (CRD42024531877). This study was supported by Precision Medicine Project (J202109) of Wuxi Health Committee.

## Author contributions

**Conceptualization:** Jikai Liu, Ting Shan.

**Data curation:** Sunyu Tang, Jikai Liu.

**Formal analysis:** Sunyu Tang.

**Funding acquisition:** Sunyu Tang.

**Investigation:** Sunyu Tang.

**Methodology:** Sunyu Tang.

**Project administration:** Sunyu Tang.

**Resources:** Ting Shan.

**Software:** Sunyu Tang, Jikai Liu, Ting Shan.

**Supervision:** Jikai Liu, Ting Shan.

**Visualization:** Jikai Liu.

**Writing – original draft:** Sunyu Tang.

**Writing – review & editing:** Jikai Liu, Ting Shan.

## Supplementary Material


