# Supplementary material for: The effect of dexmedetomidine on emergence delirium of postanesthesia events in pediatric department: A systematic review and meta-analysis of randomized controlled trials
Source: Medicine (Baltimore). 2024 Sep 6;103(36):e39337. doi: 10.1097/MD.0000000000039337 (PMC11384065; doi:10.1097/MD.0000000000039337)
Supplement: Supplementary file 1 [file medi-103-e39337-s001.docx]

TABLE S1 | The number of patients with ED in DEX and comparator groups

| Article | DEX(N/total) | Comparison(N/total) |
| --- | --- | --- |

| Saline |
| --- |

| Bong 2015 | 3/40 | 2/41 |
| --- | --- | --- |
| Cao 2016 | 0/30 | 3/30 |
| Chen 2013 | 3/27 | 11/24 |
| Elghamry 2021 | 6/34 | 19/33 |
| Hadi 2015 | 5/45 | 22/47 |
| Hauber 2015 | 69/195 | 125/198 |
| Huang 2022 | 6/29 | 24/28 |
| Isik 2006 | 0/21 | 7/21 |
| Jia 2017 | 10/31 | 22/31 |
| Makkar 2016 | 3/32 | 13/32 |
| Pandey 2022 | 9/76 | 58/76 |
| Raman 2023 | 1/50 | 30/51 |
| Shama 2023 | 1/25 | 10/25 |
| Shi 2019 | 14/45 | 24/45 |
| Soliman 2022 | 2/30 | 12/30 |
| Sun 2017 | 3/25 | 9/25 |
| Tsiotou 2018 | 6/31 | 14/29 |
| Yao 2020 | 6/52 | 25/51 |
| Zhang 2022 | 5/20 | 10/20 |

| Midazolam |
| --- |

| Aydogan 2013 | 2/16 | 5/16 |
| --- | --- | --- |
| Bromfalk 2023 | 1/30 | 8/26 |
| Cho 2020 | 9/34 | 10/32 |
| Mountain 2011 | 3/22 | 5/19 |
| Ramlan 2021 | 4/32 | 9/32 |
| Wang 2020 | 0/30 | 6/30 |
| Yao 2020 | 6/52 | 22/50 |

| Propofol |
| --- |

| Bong 2015 | 3/40 | 0/39 |
| --- | --- | --- |
| Han 2022 | 0/26 | 8/27 |
| Huang 2022 | 6/29 | 17/29 |
| Makkar 2016 | 3/32 | 5/36 |

| Clonidine |
| --- |

| Bromfalk 2023 | 1/30 | 3/26 |
| --- | --- | --- |

| Dezocine |
| --- |

| Jia 2017 | 10/31 | 15/31 |
| --- | --- | --- |

| Ketamine |
| --- |

| Chen 2013 | 3/27 | 6/27 |
| --- | --- | --- |

| Esketamine |
| --- |

| xu 2022 | 0/55 | 0/56 |
| --- | --- | --- |

| Ondansetron |
| --- |

| Shama2023 | 1/25 | 7/25 |
| --- | --- | --- |

| Dexamethasone |
| --- |

| Shama 2023 | 1/25 | 4/25 |
| --- | --- | --- |

| non-drug |
| --- |

| Soliman 2022 | 2/30 | 4/30 |
| --- | --- | --- |
| Yao 2020 | 7/30 | 7/30 |
