# Supplementary material for: The effect of dexmedetomidine on emergence delirium of postanesthesia events in pediatric department: A systematic review and meta-analysis of randomized controlled trials
Source: Medicine (Baltimore). 2024 Sep 6;103(36):e39337. doi: 10.1097/MD.0000000000039337 (PMC11384065; doi:10.1097/MD.0000000000039337)
Supplement: Supplementary file 2 [file medi-103-e39337-s002.docx]

TABLE S2| The number of patients with ED in different doses of DEX and comparator groups.

| article | Saline(N/total) | DEX1(0.25μg/kg) (N/total) | DEX2（0.5μg/kg）(N/total) | DEX3（0.75μg/kg）(N/total) | DEX4（1μg/kg）(N/total) | DEX5(2μg/kg) (N/total) |
| --- | --- | --- | --- | --- | --- | --- |
| Song 2016 | 15/25 | 12/25 | 11/25 |  | 6/28 |  |
| Abdel-Rahman 2018 | 10/30 | 4/30 | 1/30 |  |  |  |
| Li 2018 | 19/30 |  |  |  | 13/30 | 9/30 |
| Chen 2018 | 6/20 | 1/20 | 1/20 | 0/20 | 0/20 |  |
| Yao 2015 | 9/29 |  |  |  | 5/30 | 3/30 |
| Lee‐Archer 2020 | 5/78 |  |  |  | 1/78(injection) | 1/78(intranasal) |
