# Supplementary material for: The effect of dexmedetomidine on emergence delirium of postanesthesia events in pediatric department: A systematic review and meta-analysis of randomized controlled trials
Source: Medicine (Baltimore). 2024 Sep 6;103(36):e39337. doi: 10.1097/MD.0000000000039337 (PMC11384065; doi:10.1097/MD.0000000000039337)

SDC Figure 1


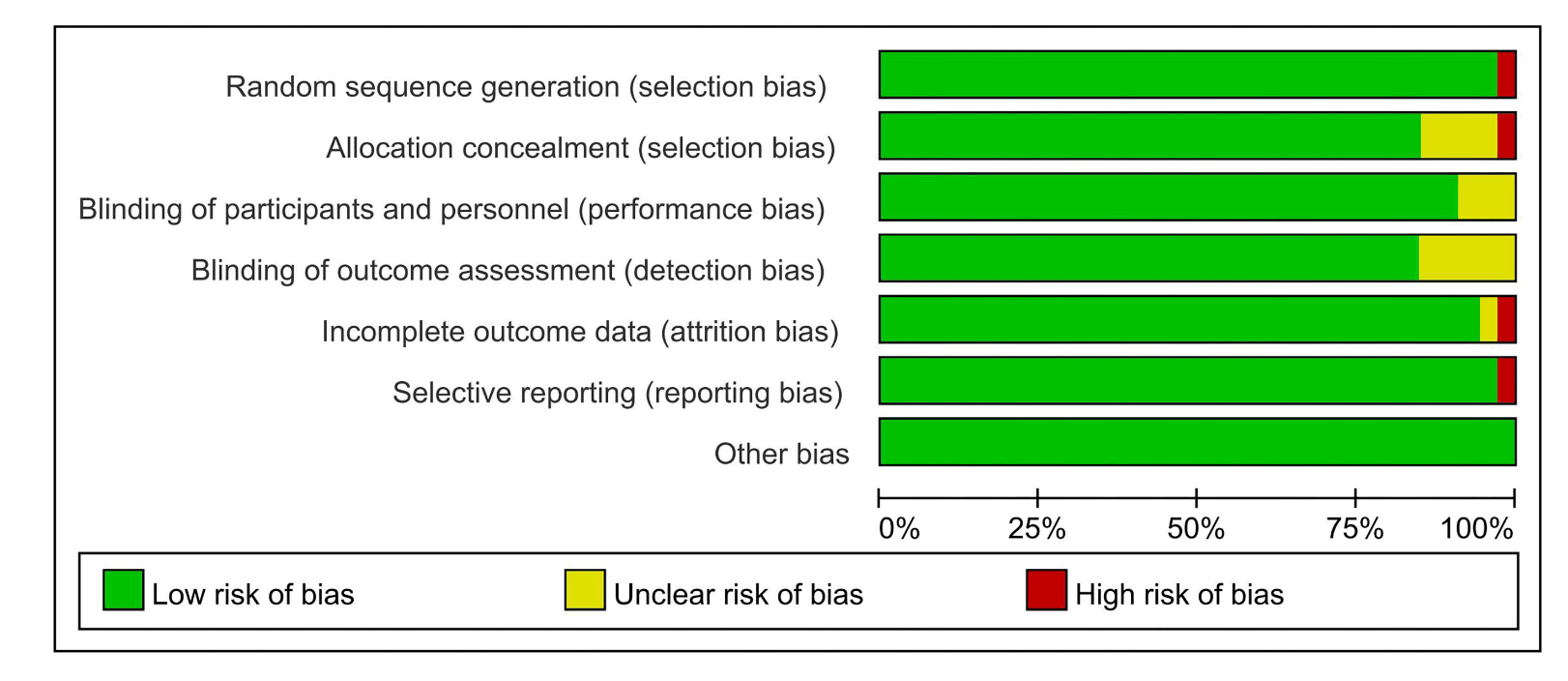


SDC Figure 2


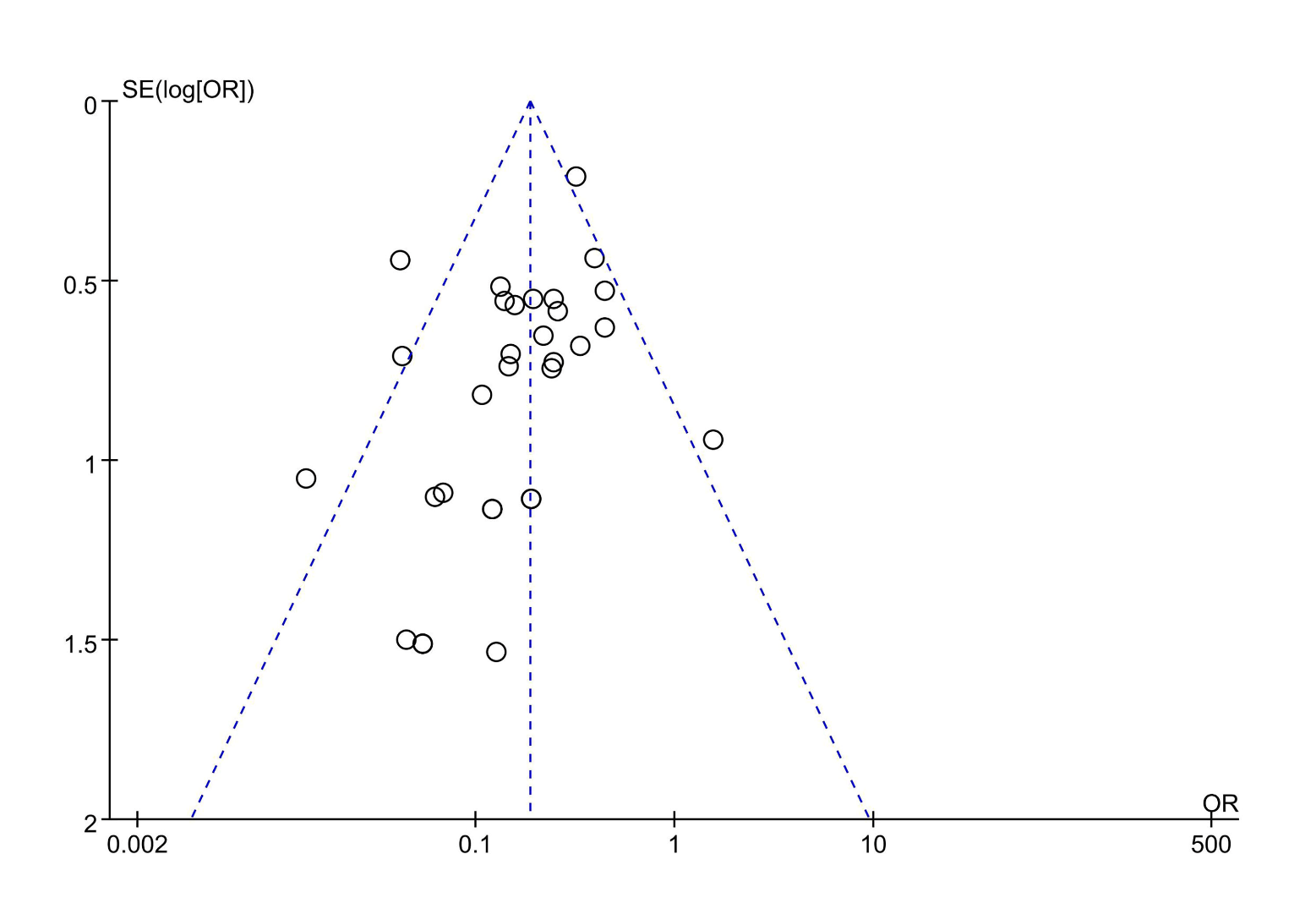


SDC Figure 3


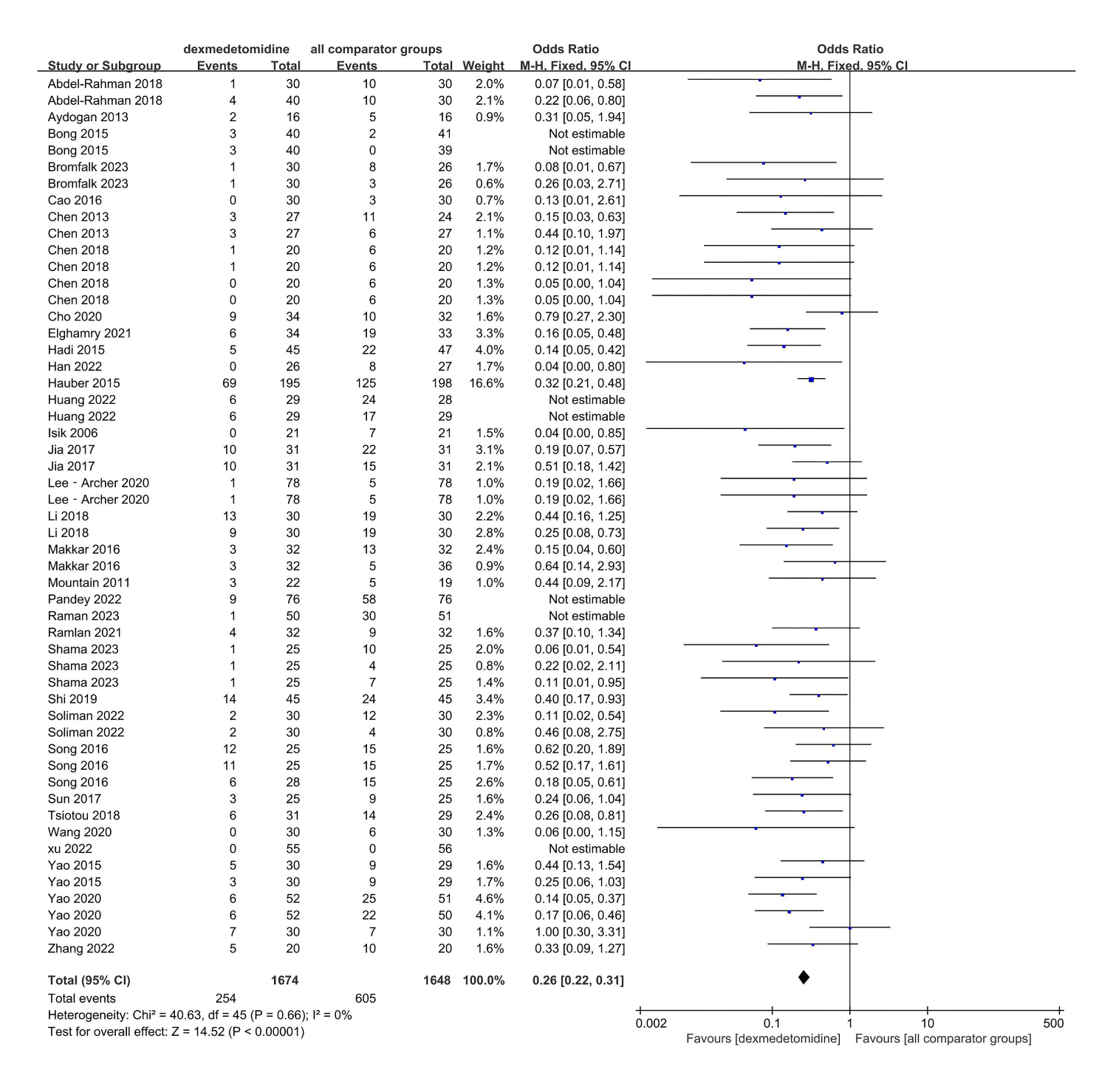


SDC Figure 4


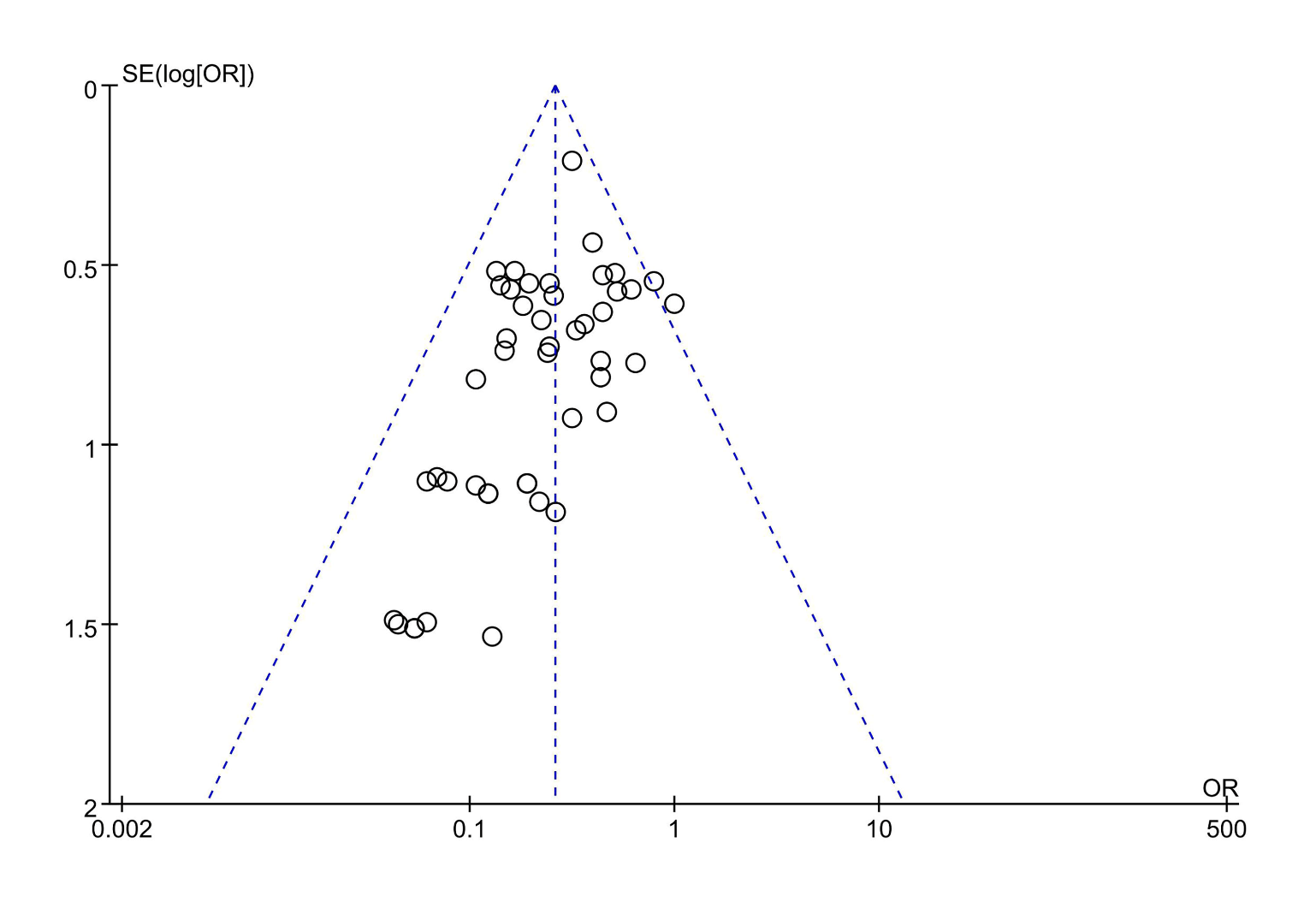


SDC Figure 5


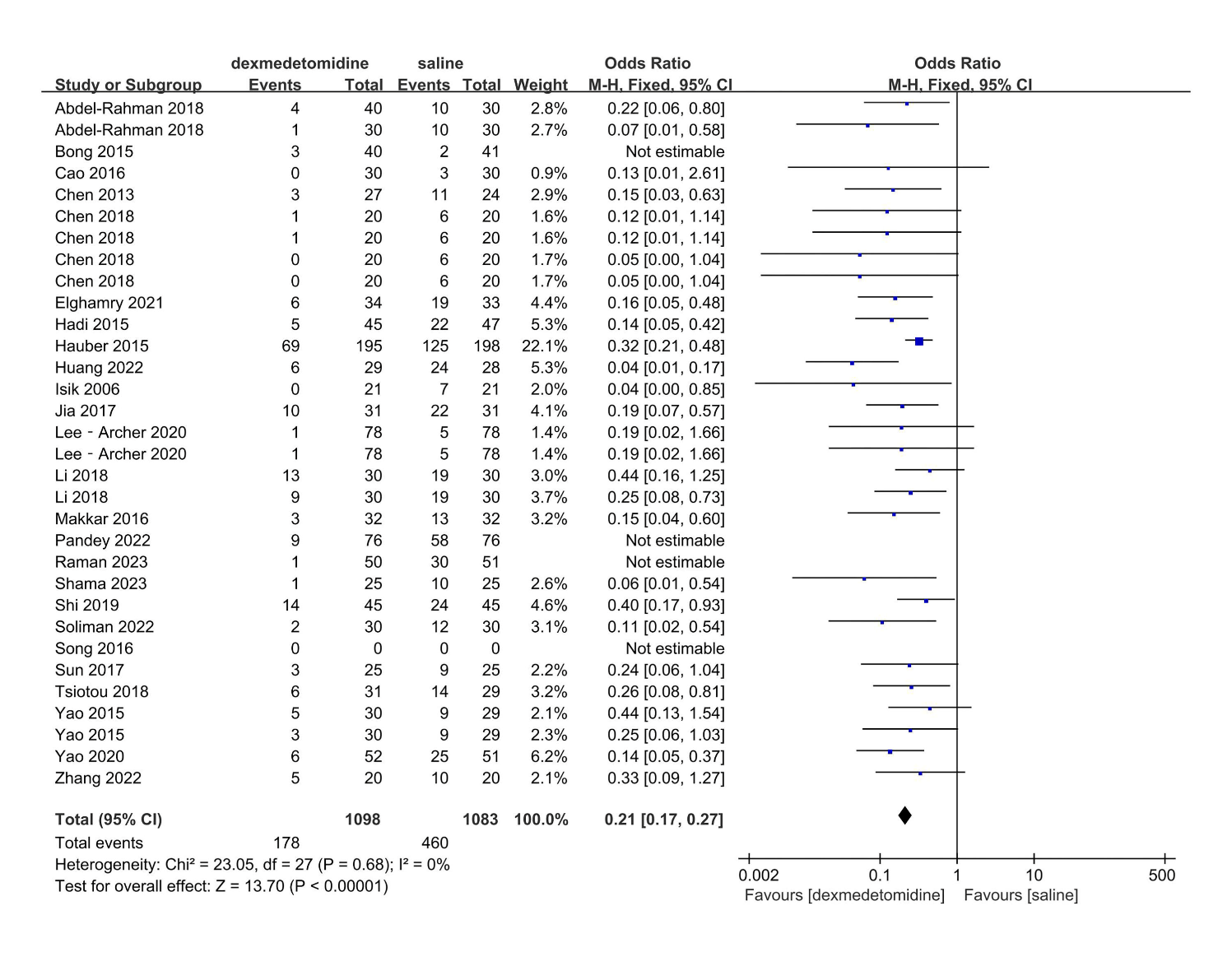


SDC Figure 6


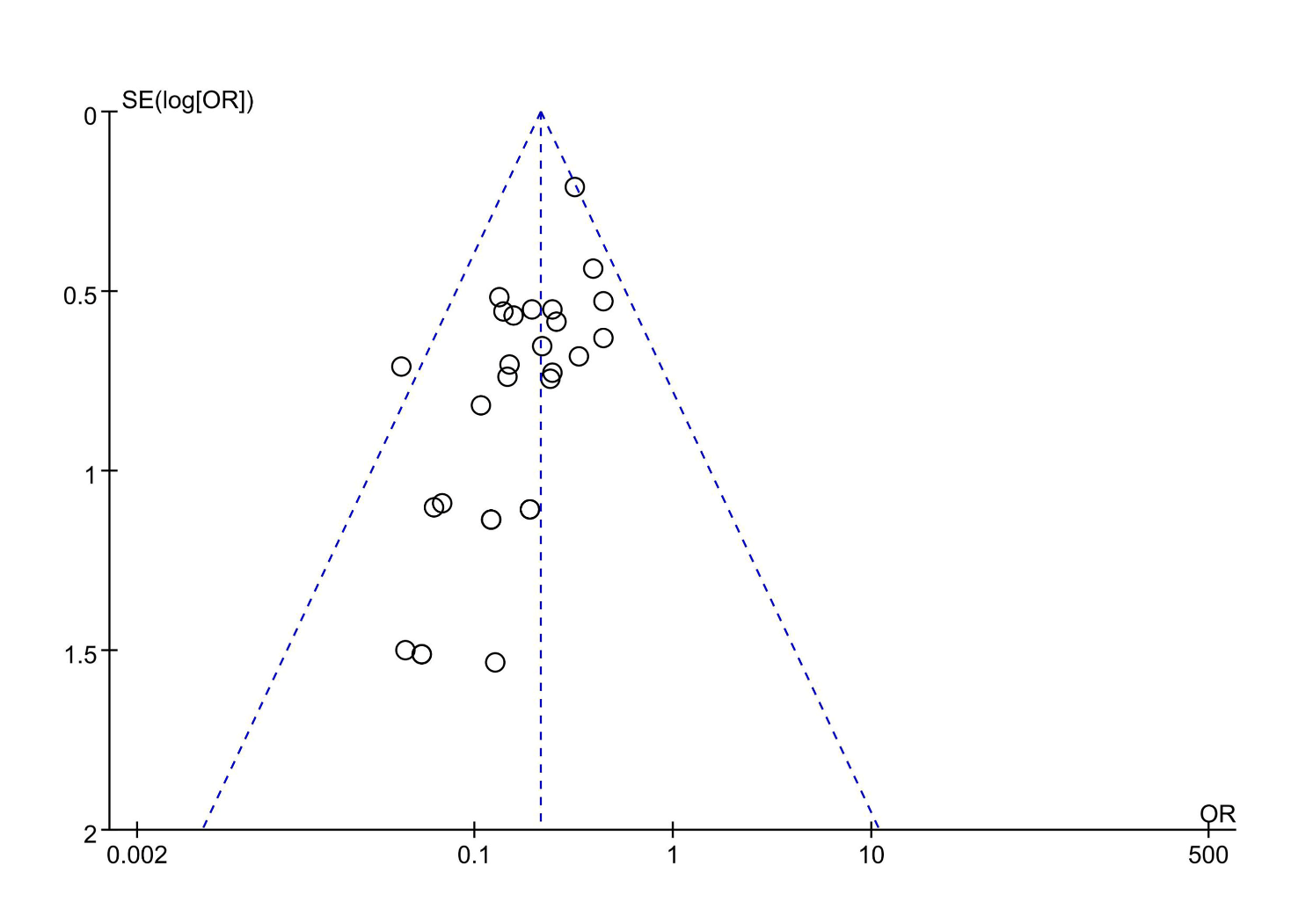


SDC Figure 7


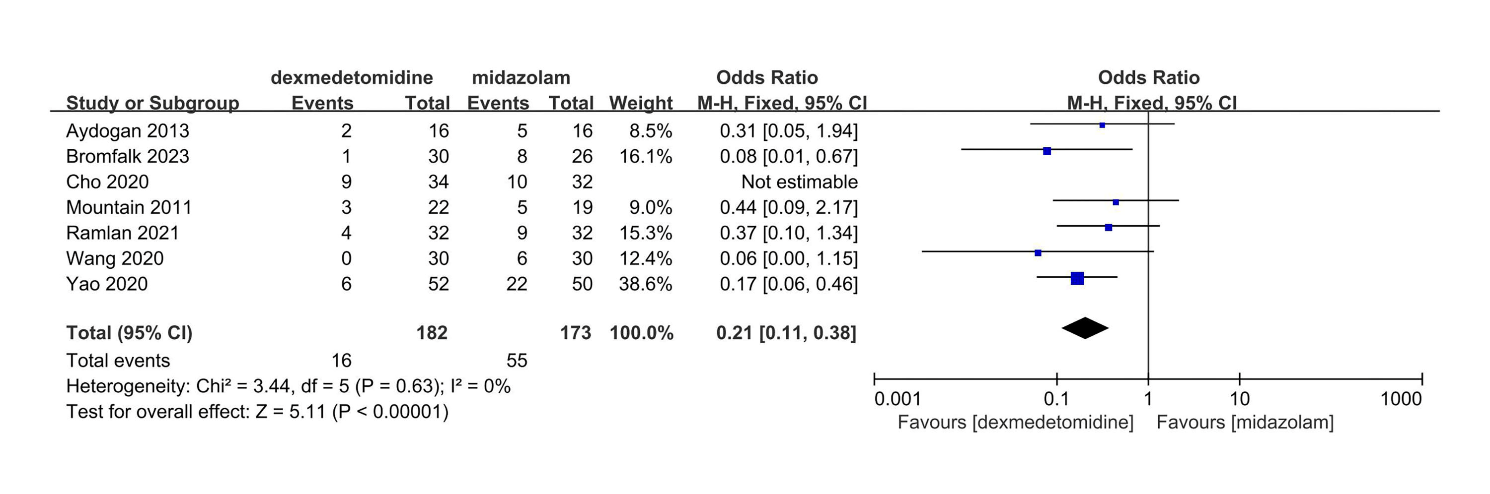


SDC Figure 8


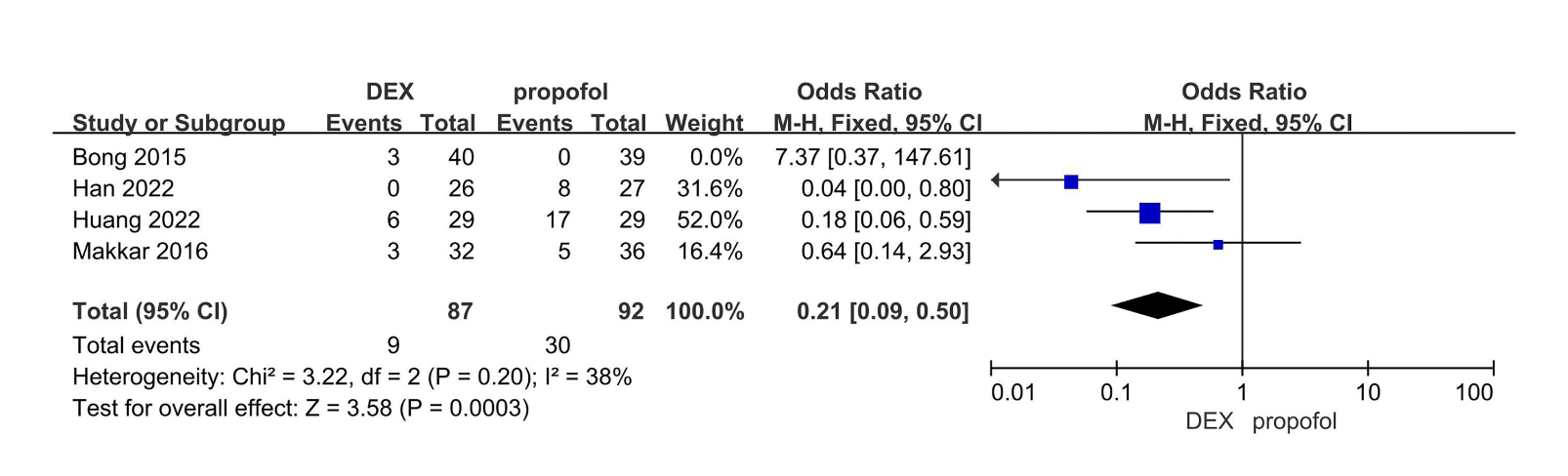


SDC Figure 9


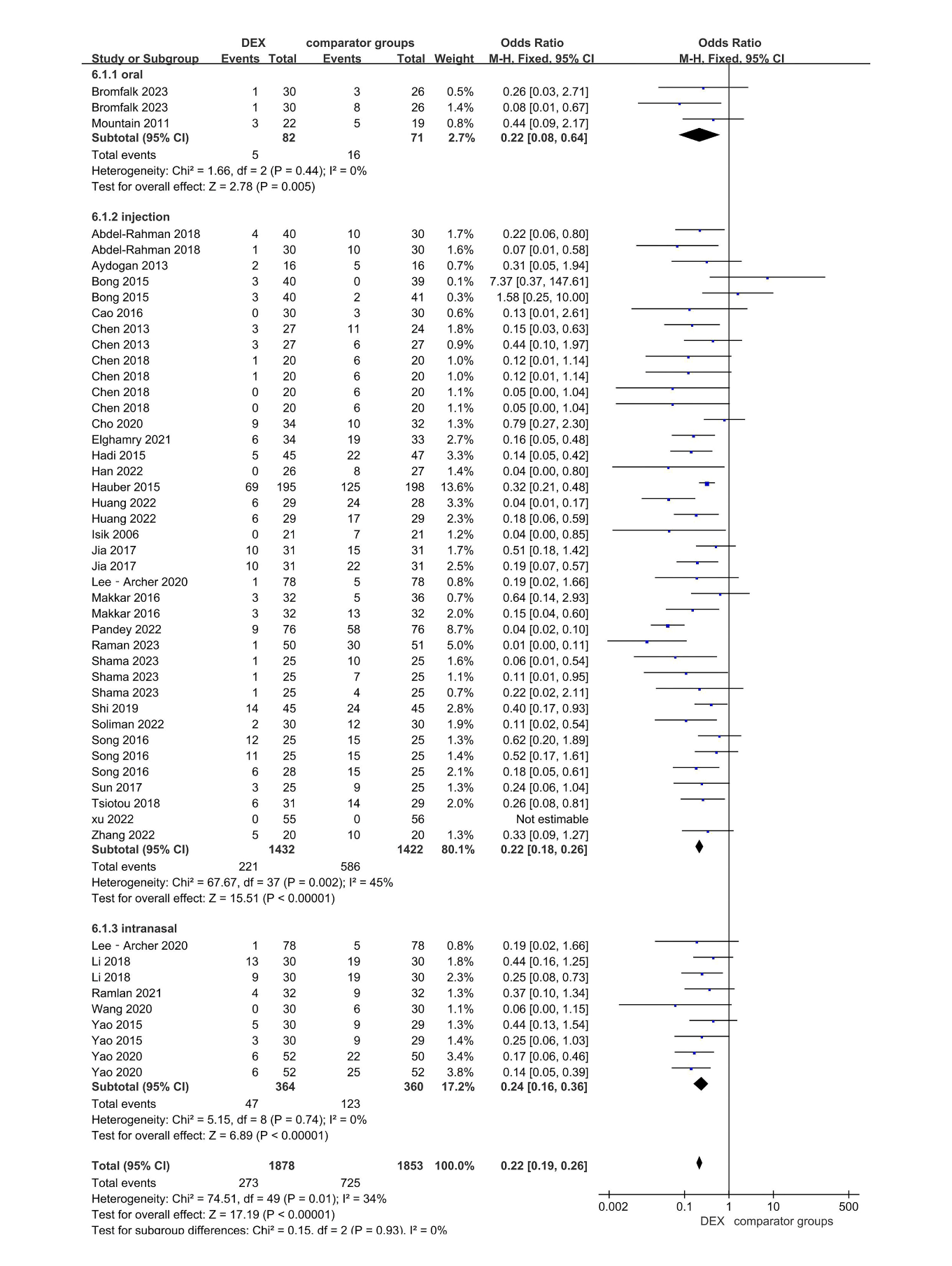


SDC Figure 10


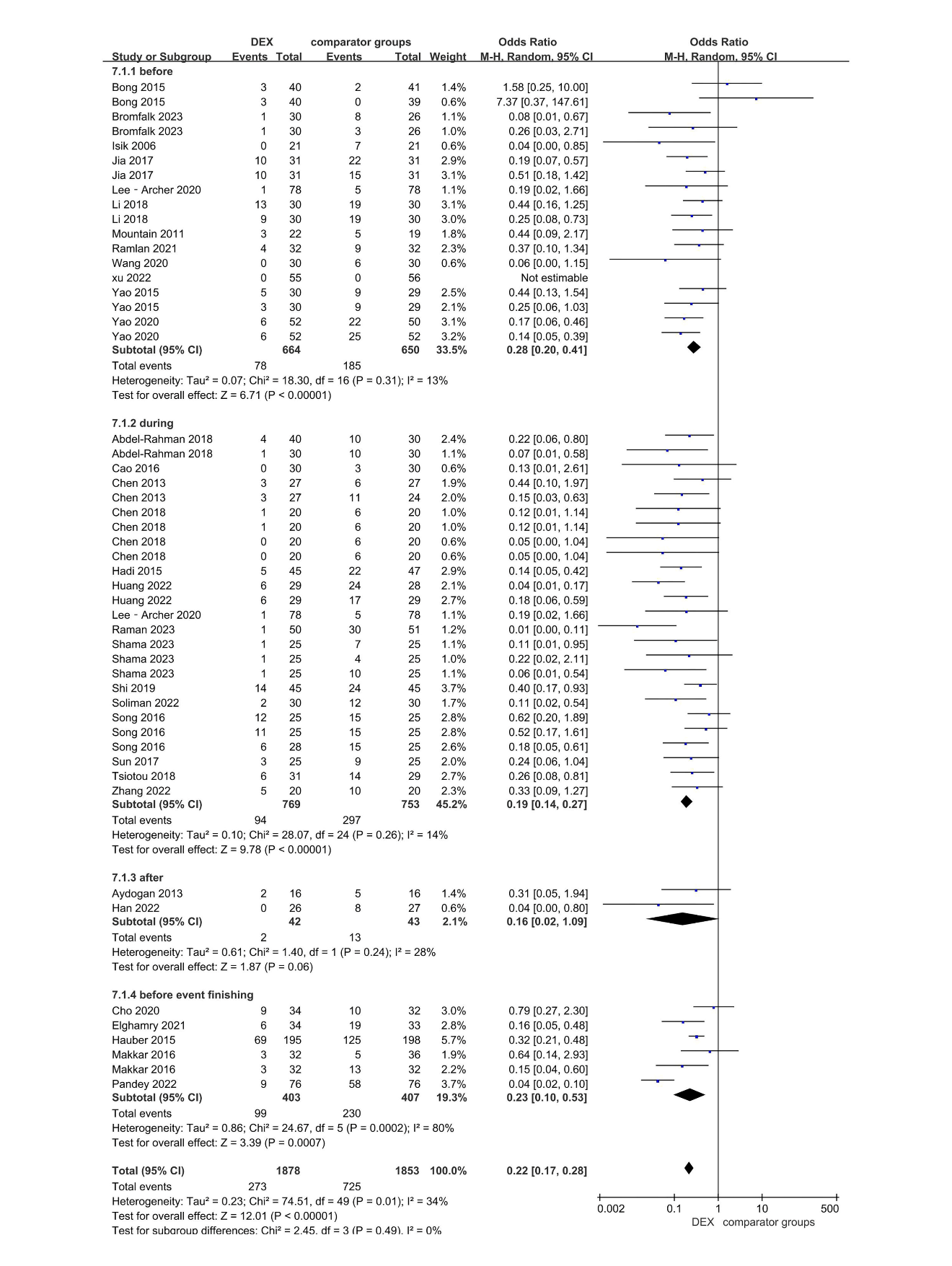


SDC Figure 11


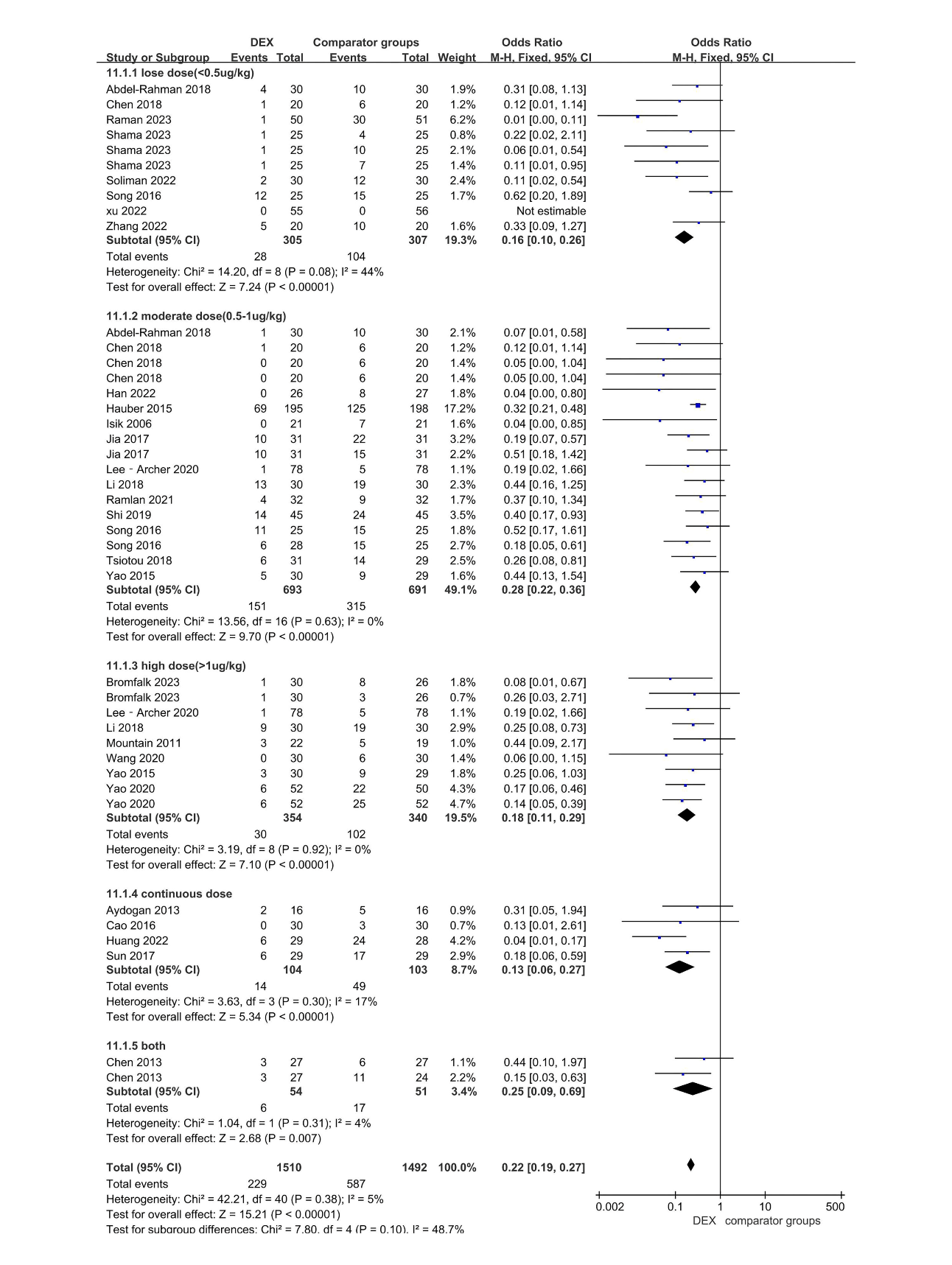


SDC Figure 12


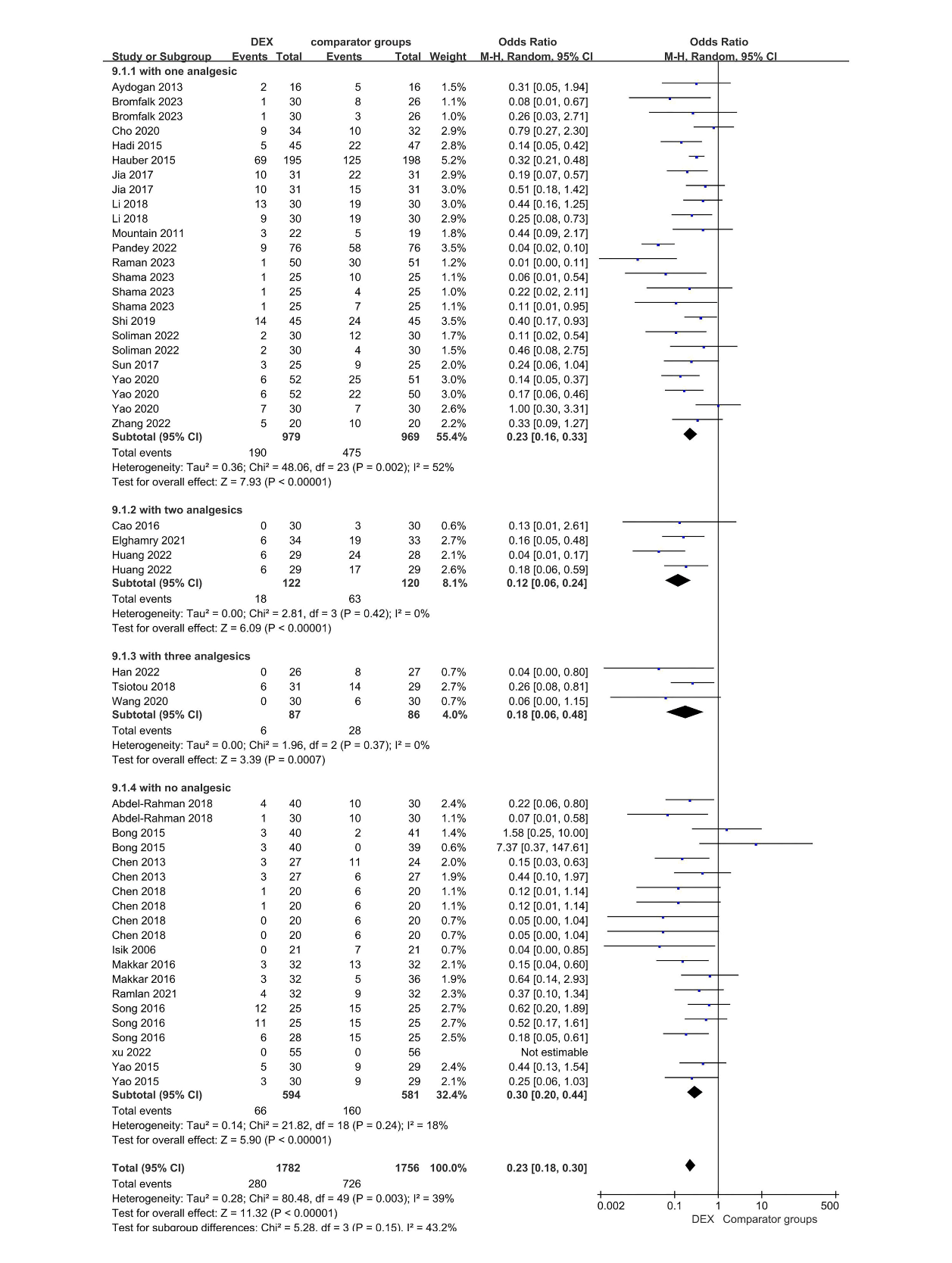


SDC Figure 13


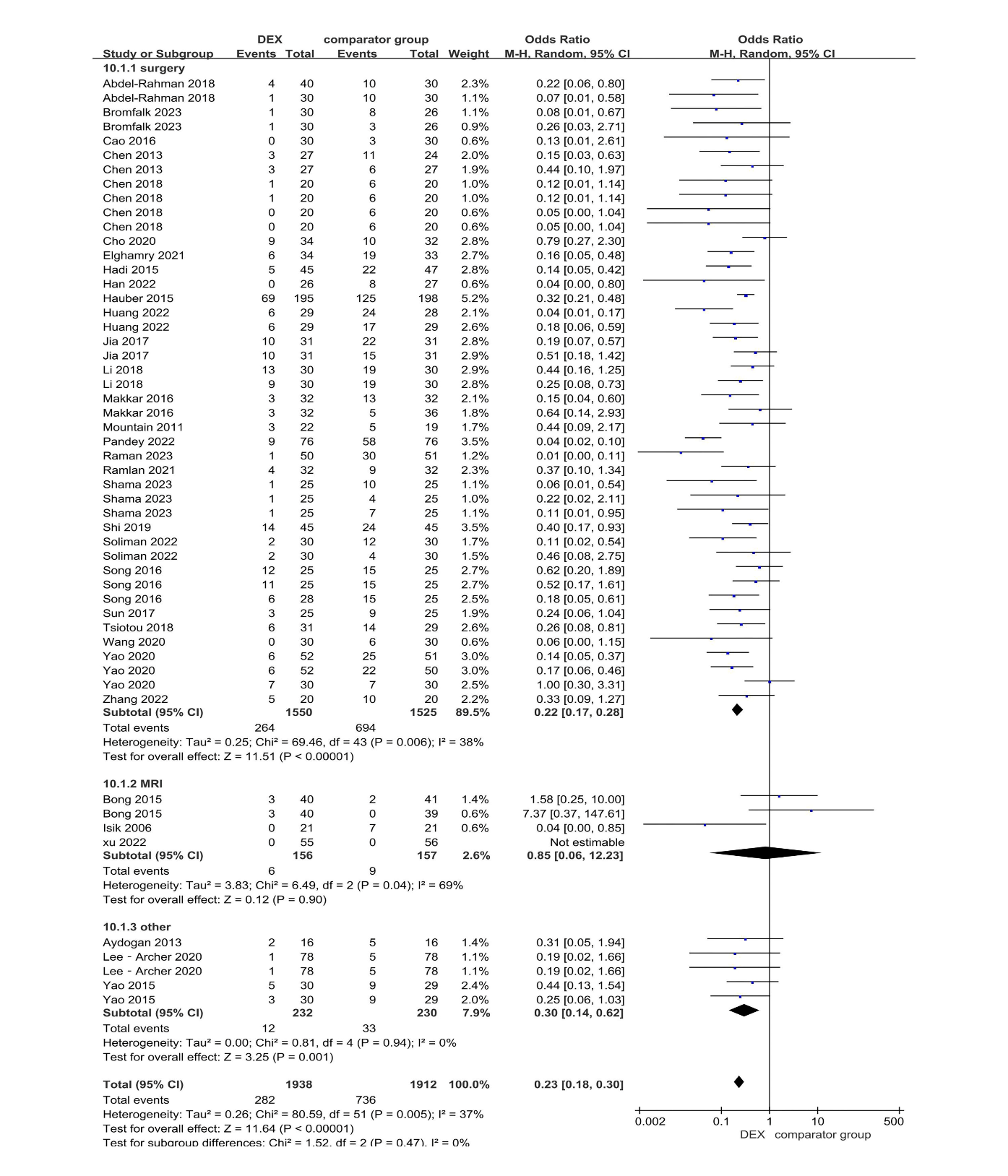


SDC Figure 14


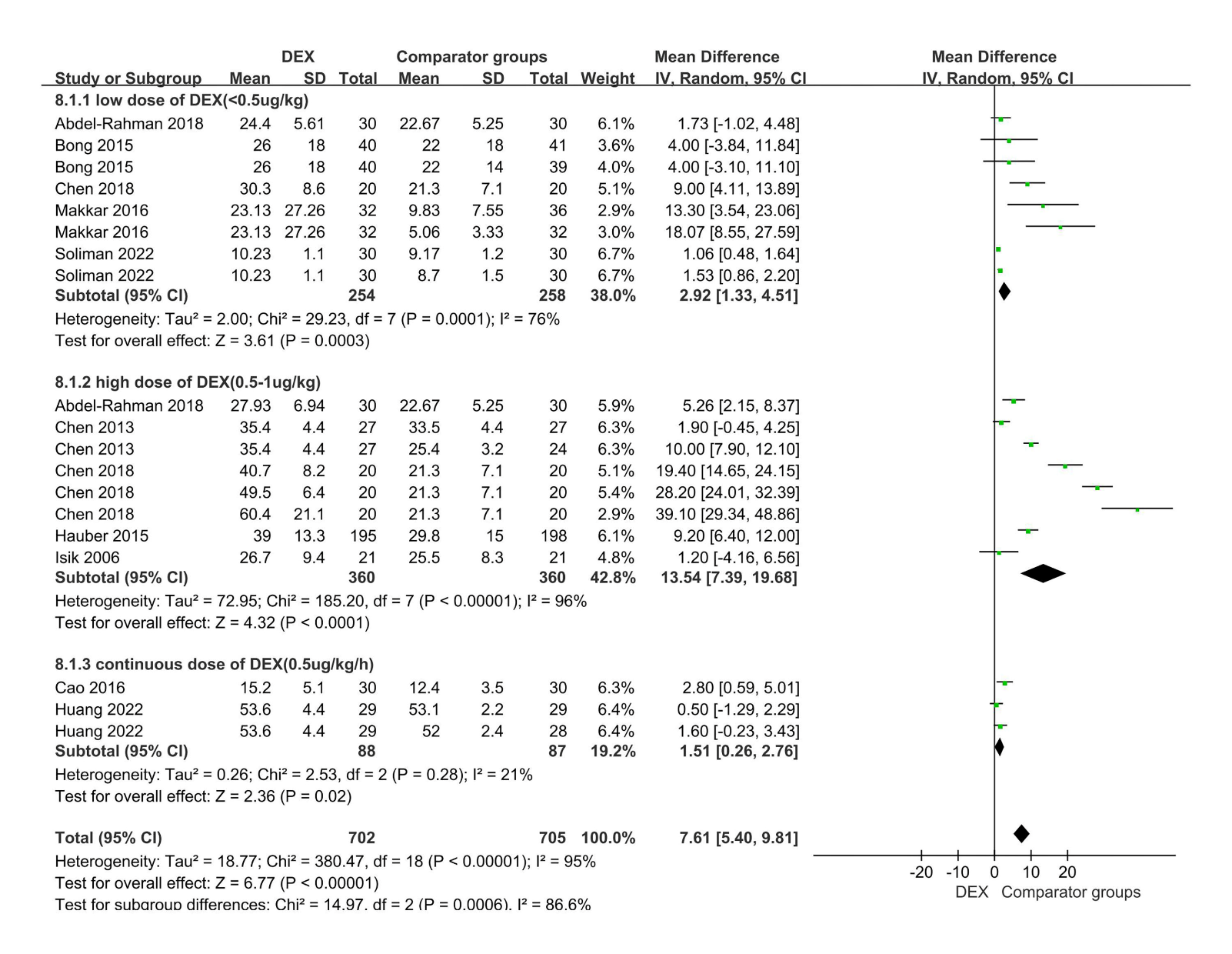

Supplement: Supplementary file 3 [file medi-103-e39337-s003.docx]
